# Comparative Microbiome and Metabolome Analyses of the Marine Tunicate *Ciona intestinalis* from Native and Invaded Habitats

**DOI:** 10.3390/microorganisms8122022

**Published:** 2020-12-17

**Authors:** Caroline Utermann, Martina Blümel, Kathrin Busch, Larissa Buedenbender, Yaping Lin, Bradley A. Haltli, Russell G. Kerr, Elizabeta Briski, Ute Hentschel, Deniz Tasdemir

**Affiliations:** 1 GEOMAR Centre for Marine Biotechnology (GEOMAR-Biotech), Research Unit Marine Natural Products Chemistry, GEOMAR Helmholtz Centre for Ocean Research Kiel, Am Kiel-Kanal 44, 24106 Kiel, Germany; cutermann@geomar.de (C.U.); mbluemel@geomar.de (M.B.); larissa.buedenbender@alumni.griffithuni.edu.au (L.B.); 2 Research Unit Marine Symbioses, GEOMAR Helmholtz Centre for Ocean Research Kiel, Duesternbrooker Weg 20, 24105 Kiel, Germany; kbusch@geomar.de (K.B.); uhentschel@geomar.de (U.H.); 3 Research Group Invasion Ecology, Research Unit Experimental Ecology, GEOMAR Helmholtz Centre for Ocean Research Kiel, Duesternbrooker Weg 20, 24105 Kiel, Germany; linyaping2012@hotmail.com (Y.L.); ebriski@geomar.de (E.B.); 4 Research Center for Eco-Environmental Sciences, Chinese Academy of Sciences, 18 Shuangqing Rd., Haidian District, Beijing 100085, China; 5 Department of Chemistry, University of Prince Edward Island, 550 University Avenue, Charlottetown, PE C1A 4P3, Canada; bhaltli@upei.ca (B.A.H.); rkerr@upei.ca (R.G.K.); 6 Faculty of Mathematics and Natural Sciences, Kiel University, Christian-Albrechts-Platz 4, 24118 Kiel, Germany

**Keywords:** biological invasion, ascidian, *Ciona intestinalis*, Prince Edward Island, microbiome, symbionts, untargeted metabolomics, bioactive secondary metabolites

## Abstract

Massive fouling by the invasive ascidian *Ciona intestinalis* in Prince Edward Island (PEI, Canada) has been causing devastating losses to the local blue mussel farms. In order to gain first insights into so far unexplored factors that may contribute to the invasiveness of *C. intestinalis* in PEI, we undertook comparative microbiome and metabolome studies on specific tissues from *C. intestinalis* populations collected in invaded (PEI) and native regions (Helgoland and Kiel, Germany). Microbial community analyses and untargeted metabolomics revealed clear location- and tissue-specific patterns showing that biogeography and the sampled tissue shape the microbiome and metabolome of *C. intestinalis*. Moreover, we observed higher microbial and chemical diversity in *C. intestinalis* from PEI than in the native populations. Bacterial OTUs specific to *C. intestinalis* from PEI included Cyanobacteria (e.g., *Leptolyngbya* sp.) and Rhodobacteraceae (e.g., *Roseobacter* sp.), while populations from native sampling sites showed higher abundances of e.g., Firmicutes (Helgoland) and Epsilonproteobacteria (Kiel). Altogether 121 abundant metabolites were putatively annotated in the global ascidian metabolome, of which 18 were only detected in the invasive PEI population (e.g., polyketides and terpenoids), while six (e.g., sphingolipids) or none were exclusive to the native specimens from Helgoland and Kiel, respectively. Some identified bacteria and metabolites reportedly possess bioactive properties (e.g., antifouling and antibiotic) that may contribute to the overall fitness of *C. intestinalis*. Hence, this first study provides a basis for future studies on factors underlying the global invasiveness of *Ciona* species.

## 1. Introduction

Biological invasions represent the second largest cause of biodiversity loss and is only surpassed by anthropogenic species extinction [1,2,3]. The marine coastal environment is one of the most invaded ecosystems globally, with at least 84% of coastal habitats affected by invasive species [4,5]. Shipping and aquaculture are considered as main vectors for the spread of marine invasive species (MIS) [4,5]. Characteristic features of MIS include high phenotypic plasticity, high fertility, and rapid growth [4,6]. MIS are often responsible for dramatic ecosystem changes, e.g., lowering species diversity, alteration of food webs and nutrient cycling [3,5], and cause drastic economic losses in various industrial sectors [5,7]. Several common invasion hypotheses have been applied to understand mechanisms allowing certain marine organisms to become invasive [7]. The prominent “enemy release hypothesis” states that invasive species thrive in their newly colonized habitats by escaping from specialized predators [1]. The “evolution of increased competitive ability hypothesis” assumes that due to lowered predation-pressure, invasive species reduce expensive specialized chemical defenses and reallocate these vacant resources towards growth and reproduction [7,8]. In the marine realm, re-distribution of resources has been suggested for the brown alga *Sargassum* spp., since herbivores preferably graze on seaweeds from the invaded population [7,9]. According to the “novel weapon hypothesis”, invasive species produce defensive metabolites conferring a competitive advantage to the invader and a potentially negative impact to native congeners [10]. 1,1,3,3-tetrabromo-2-heptanone, a secondary metabolite produced by the invasive red alga *Bonnemaisonia hamifera,* inhibits the settlement of indigenous algae [11] and is therefore an impressive example for this hypothesis. Chemical defense is often provided or aided by microbial symbionts, highlighting the importance of microbiome in invasion [12]. The “biological weapon hypothesis” postulates a transport of potential microbial pathogens and parasites by the invasive species to new habitats [13]. For instance, the crayfish *Pacifastacus leniusculus* transported the fungal pathogen *Aphanomyces astaci* to Europe, which significantly reduced native North Sea crayfish populations [13,14].

Tunicates are sessile filter-feeders with a protective outer coating, the tunic [15,16]. The fully marine tunicate class Ascidiacea is one of the most invasive marine taxa worldwide and therein *Ciona intestinalis* and its sister species *C. robusta* are among the most notorious invasive ascidians [4,17]. The sea vase *C. intestinalis* is native to the NE Atlantic and its adjacent seas (North and Baltic Seas), where it occurs in moderate abundances [17,18]. In recent years, *C. intestinalis* has spread globally and is generally regarded as a pest in invaded habitats in the NW Atlantic and Bohai and Yellow Seas (China) [17,18]. *Ciona intestinalis* reportedly reduces species richness of the sessile macrobiota in invaded habitats [19]. It is also a successful macrofouler on mussels cultivated on long lines and directly competes for food and space [17,20]. Fouling by *C. intestinalis* causes high mussel mortalities (up to 50%), leading to significant economic losses for the aquaculture industry [16,21]. Its substantial economic impact is well demonstrated in Prince Edward Island (PEI, Canada). Here, the tunicate was first observed in 2004 and rapidly became the most problematic fouling species [20]. PEI’s aquaculture economy is particularly compromised by invasive ascidians, since it produces >80% of all Canadian farmed mussels, accounting for an economic value of approx. Can $28 million per annum [22]. Together with the above outlined characteristics of MIS, *C. intestinalis*’ adaptive capacity (eurytherm, −1–35 °C, and euryhaline, 12–40‰) is considered as a major factor for its invasiveness [4]. Genetic admixture and epigenetic modifications are also regarded as promotive factors for rapid acclimation and the trans-Atlantic spread of *C. intestinalis* [23,24]. However, factors promoting the invasiveness of *C. intestinalis* are still not fully understood.

Current evidence suggests a prominent role for defensive secondary metabolites and associated (potentially pathogenic) microbiota in globally successful MIS [10,13,25]. Herein, we aimed at gaining first insights into their potential roles for the invasiveness of *C. intestinalis*. Therefore, integrated metabolome and microbiome studies on *C. intestinalis* specimens from native (Helgoland, Germany, North Sea and Kiel, Germany, Baltic Sea) and invaded (PEI, Canada, Gulf of Saint Lawrence) habitats were performed. The microbiome of the tunic and the gut was characterized, comparatively, by amplicon sequencing of the V3-V4 hypervariable region of the 16S rRNA gene. Likewise, the tunic and inner body of individual *C. intestinalis* specimens were comparatively profiled by a UPLC-MS/MS-based (ultra-performance liquid chromatography-tandem mass spectrometry) untargeted metabolomics approach. Furthermore, the global metabolome of the three ascidian populations was investigated by untargeted metabolomics using state-of-the-art tools (global natural products social molecular networking (GNPS) dereplication workflow [26], in-silico prediction [27], molecular networking (MN) [26]).

## 2. Materials and Methods

### 2.1. Sampling

Sampling of native *C. intestinalis* specimens was conducted in September 2017 in Helgoland (Germany, North Sea; 54°10′37.6″ N 7°53′35.0″ E; <1 m depth) and Kiel Fjord (Germany, Baltic Sea; 54°22′55.4″ N, 10°94′3.4″ E; ca. 3 m depth). Invasive specimens were sampled in PEI (Canada, Atlantic Ocean; 46°10′12.8″ N 62°33′52.1″ W; ca. 2 m depth) in October 2017. Seawater samples (1 L each) were collected aseptically in triplicate. Ascidian and water samples were immediately transported to the laboratory and processed at the same day. For individual genetic and chemical analyses of *C. intestinalis*, 10 intact individuals were chosen per sampling location, transferred into sterile 50 mL reaction tubes and promptly frozen at −80 °C. For chemical extractions at population level, approximately 0.5 kg of ascidian material were collected per sampling site in plastic bags and directly frozen at −80 °C. Seawater samples were sterile filtered as described in Parrot et al. 2019 [28] and filters were stored until further processing at −80 °C. For genotyping and microbiome analysis, individual animals were briefly thawed and dissected under sterile conditions. To ensure reproducibility, all dissections were done by the same person. First, the tunic was separated from the mantle and the remaining body. An approximately 4 cm^2^ piece of the tunic was cut for extraction of microbial DNA. Subsequently, the gut was removed from the inner body and the gut content was removed by flushing it with sterile ultrapure water (sterile filtered and UV-treated) by using an injection needle attached to a syringe. A subsample of the gut (0.04 cm^2^), which was stored at −20 °C until further processing, was taken for genotyping the *C. intestinalis* individuals. From individuals with a very short gut (individuals CT4, KT3-5), a small part of the tunic was frozen for genotyping instead. Another ca. 2 cm of gut tissue was immediately subjected to microbial DNA extraction. For individual metabolomics analysis, the remaining gut, mantle, and inner body tissues (hereinafter referred to as “inner body”) were placed into a sterile 50 mL reaction tube and the remaining tunic into a sterile 15 mL reaction tube. Samples were stored at −80 °C until metabolomic analysis.

### 2.2. Genotyping

To validate the taxonomic identification of *C. intestinalis* for all three sampling locations, 30 individuals (10 per sampling site) were genotyped with the mitochondrial marker fragment COX3-ND1 [29]. Genomic DNA was extracted from gut or tunic tissue using the proteinase K method [30]. Amplification of the target DNA fragment was performed with the primers TX3F and TN1R [29] in 25 µL reactions containing approximately 100 ng template DNA, 1 U TaKaRa Ex Taq (Takara, Dalian, China), 2.5 µL of 10X Ex Taq buffer and 200 µM dNTP mixture. Besides an increased elongation time of 45 s, PCR conditions were applied as previously described by Zhan et al. 2010 [31]. The target band (ca. 600 bp) was purified from a 1% TBE gel by using purification beads (iCloning Beijing Biotech, Beijing, China). Subsequent Sanger sequencing was performed with the primer TX3F on an ABI 3130*xl* capillary sequencer. Sequences were aligned in BioEdit [32] using the ClustalW [33] multiple alignment tool. Phylogeny was inferred by constructing a Maximum Likelihood tree based on the General Time Reversible model [34] in MEGA7 [35]. All collected specimens were identified as *C. intestinalis* (Figure S1).

### 2.3. Microbiome Analysis

#### 2.3.1. DNA Extraction, Library Preparation, and Sequencing

For microbial community composition analyses, genomic DNA was extracted from tunic (*n* = 30), gut (*n* = 30), and seawater samples (*n* = 9; Table S1) using the DNeasy PowerSoil Kit (Qiagen, Hilden, Germany). A piece of the respective tissue (gut: approximately 1 cm in length, tunic: approximately 2.5 × 1.5 cm) or a piece of the filter equivalent to 250 mL seawater reference sample was transferred into a provided PowerBead Tube containing 60 µL of solution C1. The tubes were shaken for 20 min at a frequency of 30/s in a mixer mill MM 200 (Retsch, Hahn, Germany). All subsequent steps were performed according to the manufacturers’ instructions besides the final step. In order to increase the DNA concentration, 30 µL instead of 100 µL of solution C6 were added to the column. The quantity of the extracted DNA was checked with a NanoDrop. The V3-V4 hypervariable region of the 16S rRNA gene was amplified with the primer pair 341F/806R [36] using the following thermal cycling conditions: 30 s 98 °C; 30 cycles of 9 s 98 °C, 30 s 55 °C, 30 s 72 °C; 10 min 72 °C. Amplified DNA fragments were separated by agarose gel (1%) electrophoresis and amplicons of the expected size (ca. 465 bp) were excised from the gel, and the DNA purified via gel elution (QIAquick Gel Extraction Kit, Qiagen), normalized, and pooled. Sequencing was performed on an Illumina MiSeq platform (MiSeqFGx) using the Illumina MiSeq Reagent Kit v3 (2 × 300 bp). Demultiplexing was performed based on 0 mismatches in the barcode sequences. Raw amplicon sequences were deposited in the Sequence Read Archive of NCBI (BioProject: PRJNA635604).

#### 2.3.2. Bioinformatic Processing and Statistical Analysis

Primer and adapter trimming of demultiplexed raw sequences was done with Cutadapt v. 2.3 [37]. This was followed by quality filtering using BBDuk [38]. Trimmed and quality filtered raw sequence reads were further processed with mothur v. 1.42.0 [39] by applying a modified version of the established MiSeq SOP [40]. Briefly, contigs containing ambiguous bases or homopolymers >8 bp were removed. Filtered contigs were aligned to the SILVA database (release 132; [41]). Subsequent filtering steps removed unaligned contigs, gap-only columns, and singletons. Contigs were preclustered as suggested by Huse and co-workers [42] and non-bacterial sequences were removed by taxonomic sequence classification using the Wang et al. method [43] at a bootstrap threshold of 80%. After eliminating chimeric sequences with the UCHIME algorithm [44], contigs were binned at a 97% similarity level into operational taxonomic units (OTUs). OTUs were classified with the 16S rRNA gene SILVA reference alignment file. Two replicates from Helgoland (HG6, HG8) and two replicates from Kiel (KG10, KT6) were removed from the dataset since the number of sequence reads was extremely low (3–118 reads).

Subsequent data analysis was done with R v. 3.5.2 [45] using the packages *phyloseq* [46], *vegan* [47], and *picante* [48]. The OTU abundance table was rarefied based on the maximum number of 3647 sequences common to all samples. Alpha diversity was estimated by calculating OTU richness, Chao1, Faith’s phylogenetic diversity (Faith’s PD), Simpson, and Shannon indices. Alpha diversity indices were statistically compared across sampling locations and sample types via one-way ANOVA and Tukey’s honest significance difference (HSD) test by using the *aov()* and *TukeyHSD()* functions in R. In order to compare the microbial diversity among sample types, non-metric multidimensional scaling (nMDS) was performed on OTU counts, which were rarefied and square root transformed for standardization (Bray-Curtis similarity index). In order to consider phylogenetic relatedness of OTUs, an additional nMDS plot based on weighted UniFrac distances [49] was calculated. Statistical testing was done by analysis of similarity (ANOSIM) calculations executed in Past v. 3.12 [50]. The Marker Data Profiling workflow offered by the platform MicrobiomeAnalyst [51] was used for detection of bacterial taxa that differed significantly between the sample types and sampling locations. Briefly, the original OTU abundance table was rarefied to the minimum library size. Significantly different bacterial taxa were statistically identified via the Kruskal-Wallis-Test (comparison of multiple groups). P values were adjusted for multiple testing using the false discovery rate (FDR) method as suggested by Benjamini and Hochberg [52].

### 2.4. Metabolome Analysis

#### 2.4.1. Solvent Extraction

For individual metabolome analyses, single ascidian specimens were dissected into inner body and tunic (*n* = 60, i.e., 10 replicates per sampling site and tissue; Table S2). Freeze-dried inner bodies and tunics were separately ground. Solvent extractions were performed by maceration in a 1:20 (solid:liquid) ratio in 3 cycles over 24 h (2 × 4 h, 1 × overnight) in the dark at 120 rpm and 22 °C. First, samples were extracted with ultrapure water in order to remove sea salts. Between the cycles and after the last extraction step, the water was separated from the cell material via centrifugation (4500 rpm, 7 min, Beckmann J2-21M centrifuge, Beckman Coulter, Brea, CA, USA). Prior to solvent extraction, the leftover cell material was again freeze-dried. Subsequent solvent extraction was done with methanol (MeOH) and dichloromethane (DCM; both purchased at AppliChem, Darmstadt, Germany). The extraction procedure was similar to the water extraction described above; the solvent was however removed by decantation instead of centrifugation. MeOH and DCM extracts were combined, filtered through a 0.2 µM PTFE syringe filter (Carl Roth, Karlsruhe, Germany), and evaporated to dryness on a rotary evaporator. Two blank extractions processed in the same manner served as control samples for UPLC-MS analysis. Organic extracts were combined and evaporated to dryness. The pooled ascidian bulk samples (i.e., approximately 500 g of fresh whole ascidians per sampling site) for population analyses were extracted similarly, using 13.0 g of freeze-dried samples (*n* = 3 per sampling site).

#### 2.4.2. LC-MS/MS Analysis and Data Pre-Processing

All solvents used for the LC-MS analyses were ULC-MS grade from Biosolve Chimie (Dieuze, France) and ultra-purified water was prepared with an Arium Lab water system (Sartorius, Goettingen, Germany). Prior to UPLC-QToF-MS/MS measurements, extracts were diluted with MeOH to a final concentration of 0.1 mg/mL and blank MeOH samples were used as solvent controls. Analyses were performed with an Acquity UPLC I-Class System coupled to a Xevo G2-XS QTof Mass Spectrometer (Waters, Milford, MA, USA), which was equipped with an Acquity UPLC HSS T3 column (High Strength Silica C18, 1.8 μm, 2.1 × 100 mm, Waters) operating at 40 °C. The injection volume was 5.0 µL. A binary mobile phase system (A: 0.1% formic acid in ultra-purified water, B: 0.1% formic acid in acetonitrile) was pumped at a flow rate of 0.6 mL/min by applying the following gradient (% of A given): initial, 99%; 2.5 min, 50%; 12.5 min, 0%; followed by washing and reconditioning of the column. The total run time was 16 min. The MS and MS/MS spectra were recorded in positive mode as previously described by Parrot et al. [28].

Recorded data were converted with msconvert [53] to mzXML format. Data pre-processing was conducted with MZmine v. 2.38 [54] by applying the following parameters. The mass list was compiled with a noise level of 500. Using this mass list, chromatograms were built with the following thresholds: minimum time span: 0.01 min, minimum peak height: 500, *m/z* tolerance: 0.01 (or 10 ppm). Chromatogram deconvolution was done with the baseline cut-off algorithm at a baseline level of 500, a minimum peak height of 1000 and a peak duration range of 0 to 0.5 min. Grouping of isotopic peaks was performed by applying the isotopic peaks grouper algorithm with the following parameters: *m/z* tolerance: 0.01 (or 10 ppm), retention time (R_t_) tolerance: 1.0 min, maximum charge: 3. In a final step, all samples were combined into one peak list by using the join aligner algorithm (*m/z* tolerance: 0.01 (or 10 ppm), R_t_ tolerance: 0.5 min, weight *m/z*:R_t_: 75:25). Compounds detected in MeOH or extraction blanks were removed from this list. The resulting data were saved in the GNPS compatible data format MGF and the comprehensive peak list was exported to Microsoft Excel 2010 for further analysis. The peak area of population level extracts was used to investigate whether a peak was homogenously distributed across all sampling locations or showed an enhanced (at least 10-fold increase compared to other sampling sites) or exclusive production at one sampling site.

#### 2.4.3. Molecular Networking and Dereplication

Pre-processed MS/MS data were submitted to the Feature-Based Molecular Networking (FBMN) workflow available at the online platform GNPS [26]. Briefly, consensus spectra were constructed with a parent mass and MS/MS fragment ion tolerance of 0.02 Da. FBMN were created with edges filtered to have a cosine score above 0.5 and more than 6 matched peaks (4 for individual metabolomes). The remaining parameters were set as previously described by Parrot et al. [28]. The FBMN was visualized with Cytoscape v. 3.7.1 [55] and nodes were color-coded by a presence-absence matrix. For automated dereplication, files were subjected to the dereplication workflow of GNPS, which was combined with the in-silico MS/MS database-based workflow of the Universal Natural Product Database (ISDB-UNPD) [27]. In addition, MS chromatograms were manually inspected and putative molecular formulae were predicted by MassLynx v. 4.1 (Waters). Putative molecular formulae were searched for known chemical entities in common natural products databases (Dictionary of Natural Products (DNP): http://dnp.chemnetbase.com, MarinLit: http://pubs.rsc.org/marinlit/ and Reaxys: https://www.reaxys.com). Putative hits were validated by biological origin. The detected fragmentation pattern was verified with the in-silico fragmentation tool CFM-ID [56] for putatively identified compounds.

#### 2.4.4. Statistical Analysis

The average yields of population level extracts were statistically compared across the three different sampling locations via one-way ANOVA and a subsequent Tukey’s HSD test as described above. For statistical comparison of the metabolite profiles at individual (*n* = 10 per sampling site and tissue) and population extract level (*n* = 3 per sampling site), nMDS plots were compiled (Bray-Curtis similarity index). Peak areas of individual level extracts were normalized by dry weight (Table S3). Significance of observed clusters (location and tissue) was tested with ANOSIM (Bray-Curtis similarity index). Finally, a Mantel test was performed using the R package *vegan* in order to test correlation of individual tunic microbiomes and metabolomes (Bray-Curtis similarity matrices).

## 3. Results

### 3.1. Comparative Microbiome Analysis of Tunic and Gut From Invasive and Native C. intestinalis

The microbiome of *C. intestinalis* and seawater reference samples from three locations was explored by amplicon sequencing of the V3-V4 hypervariable region of the bacterial 16S rRNA gene. Sampling was conducted at an invaded (PEI, Canada, C) and two native habitats of *C. intestinalis*, i.e., Helgoland (H) and Kiel (K). From each location, three different sample types (tunic: T, gut: G, seawater samples: W) were analyzed (Table S1). Quality filtering reduced the total number of read pairs from 2443581 to 1292969 (Figure S2). Binning of raw read pairs into OTUs at a similarity level of 97% and subsequent rarefying yielded 5211 OTUs.

Across all samples, considerable proportions of singletons (26.1%) and rare OTUs (99.8% with <1% relative abundance) were detected. According to the distribution of singletons, rarefaction curves indicated sufficient coverage of gut and not yet completely saturated curves for tunic and seawater samples (Figure S3). Average OTU richness was higher in tunic (413) than in gut (247) samples (ANOVA: df: 2, F: 9.1, *p* < 0.001, Tukey’s HSD: *p* = 0.0012; Table S4). Similarly, tunic samples showed higher phylogenetic diversity (20.5) than gut samples (14.7; ANOVA: df: 2, F: 6.1, *p* = 0.003, Tukey’s HSD: *p* = 0.02). The Shannon and Simpson diversity indices were similar for both tissues (Shannon: 3.9 (G), 3.7 (T); Simpson: 0.89 (G), 0.88 (T)). Location-wise, all alpha diversity measures were on average highest for Canadian *C. intestinalis* specimens. For instance, the diversity measures were on average higher in the Canadian population (PD: 21.9, Shannon: 4.3, Simpson: 0.93) when comparing to Helgoland (PD: 12.1, Shannon: 3.4, Simpson: 0.90) and Kiel Fjord populations (PD: 18.6, Shannon: 3.7, Simpson: 0.84). One-way ANOVA verified significantly different OTU richness and PD when comparing all samples (ANOVA PD: df: 8, F: 9.3, *p* < 0.001; ANOVA OTU count and Chao1: df: 8, F: 10.0–18.6, *p* < 0.001), but was insignificant for the Shannon and Simpson diversity indices (ANOVA: df: 8, F: 1.3–2.1, *p* > 0.05). The subsequently calculated Tukey’s HSD test revealed for some compared groups significant differences, e.g., the number of detected OTUs and the PD was significantly higher in Canadian tunics compared to that of Helgoland (*p* < 0.01), while comparison to Kiel tunics was insignificant (*p* > 0.05; Table S5).

**Figure 1:**
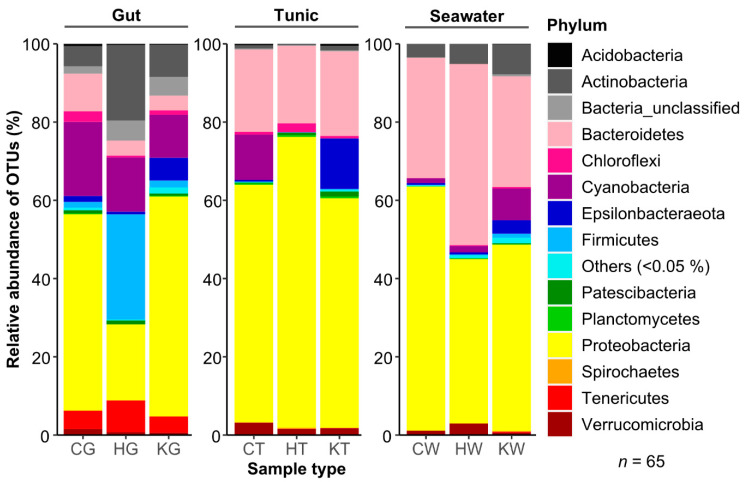
Comparative microbiome analysis of three *C. intestinalis* populations. The taxonomic assignment was conducted with SILVA (release 132) and is shown on the phylum level. Replicates were combined by calculating the median relative abundance for each phylum. Samples were abbreviated with a letter for the respective sampling site (Canada, C; Helgoland, H; Kiel, K) and sample type (gut, G; tunic, T; seawater, W).

**Figure 2:**
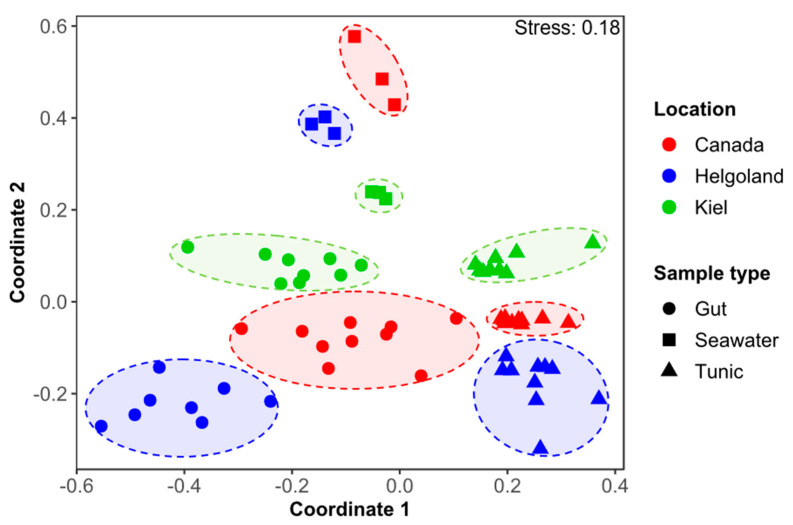
Across sample type and geographic origin comparison of the *C. intestinalis*-associated microbiome. The 2D nMDS plot was calculated using the full set of detected OTUs (5211) and is based on a Bray-Curtis similarity matrix.

Phylogenetic analysis assigned OTUs to 39 different bacterial phyla. Although differential abundances across the different samples were observed, the phyla Proteobacteria (53.8%), Bacteroidetes (16.6%), Cyanobacteria (8.5%), and Actinobacteria (5.1%) were the most abundant across all sample types (Figure 1). Multivariate ordination showed different clusters matching the nine different sample groups (i.e., three locations and three sample types; Figure 2). Hence, microbiome profiling revealed clustering by both sample type and location (Figure 2 and Figure S4). Ascidian microbiomes from all three sampling sites differed significantly from seawater reference samples (R: 0.73–0.96, *p* = 0.001), but also tunic and gut tissues had different microbiome profiles (R: 0.70, *p* = 0.001; Table S6). ANOSIM also confirmed the observed clustering by sampling site statistically (R: 0.73–0.98, *p* = 0.001). In order to verify the robustness of the beta-diversity results, we applied an additional ordination based on weighted UniFrac distances. In accordance with the nMDS plot based on the Bray-Curtis similarity index, the UniFrac-based ordination plot showed clustering by sample type and sampling location while being less apparent (Figure S5). ANOSIM on weighted UniFrac distances confirmed distinct clustering of the nine sample groups (R: 0.86, *p* < 0.001) and revealed stronger impact of the sample type (R: 0.73, *p* < 0.001) compared to the sampling locations (R: 0.24, *p* < 0.001).

In-depth analyses elucidated the main bacterial taxa with significantly differential abundance (Tables S7–S10). Gut microbiomes were dominated by Cyanobacteria, Proteobacteria, and Firmicutes (the latter only in Helgoland samples; Figure 1, Table S7). Among the most abundant OTUs, four were highly abundant in all gut samples (OTUs 6, 8, 10, 16), and some were specifically prevalent in guts from one sampling location, i.e., OTU4 (K), OTU13 (H) and OTU15 (C; Table S9). Tunic tissues from all three sampling locations were mainly colonized by Proteobacteria (mainly Alphaproteobacteria) and Bacteroidetes (Figure 1, Tables S7 and S8), which matches the dominant OTUs in tunic samples (OTUs 1, 2, 7: Alphaproteobacteria, OTU3: Bacteroidetes; Table 1, Tables S9 and S10). Comparison of the tissue-specific microbiotas showed that the gut was enriched with Actinobacteria, Cyanobacteria, Firmicutes, and Tenericutes, whereas the tunic had a higher abundance of Bacteroidetes, Proteobacteria, and Verrucomicrobia (Table S7). Tissue-specific bacterial associations were corroborated by several OTUs, showing tissue-specificity towards gut (OTUs 6, 8, 10, 16) or tunic (OTUs 1–3, 7), irrespective of the geographical origin (Table 1, Table S9). Finally, OTU2 (unclassified Rhizobiales) and OTU6 (*Synechococcus* sp.) were present in all tunic and gut samples and are hence regarded as core OTUs (Table 1, Table S9 and S10). Notably, among the abundant bacterial OTUs (≥1%) that showed significantly different abundances, six OTUs were previously isolated from the gut (OTUs 7, 10, 16) or tunic (OTUs 1–3) of *C. intestinalis*, which matches with the dominant sample type determined in this study for the abundant OTUs.

With regard to the geographic location, the phylum Epsilonbacteraeota was specifically associated with all Kiel samples (e.g., OTUs 5, 20, 24 assigned to *Arcobacter* sp.), while Helgoland specimens showed a higher proportion of Actinobacteria (e.g., *Bifidobacterium* OTU13) and various Firmicutes (e.g., OTUs 36, 39 47, 50, 68) in their gut (Tables S7–S9). Cyanobacteria (e.g., *Leptolyngbya* sp. OTU14 (*Acrophormium* is the heterotypic synonym of *Leptolyngbya*) and unclassified Oxyphotobacteria OTU80) and Alphaproteobacteria (e.g., *Roseobacter* sp. OTU15, *Ruegeria* sp. OTU106, and unclassified Rhodobacteraceae OTUs 59 and 74) were elevated in tunics or guts from Canadian specimens, respectively. Interestingly, the cyanobacterial genus *Leptolyngbya* (e.g., OTU 14) was only detected in ascidians from PEI, while the actinobacterial genus *Bifidobacterium* was exclusive to guts of ascidians from Helgoland specimens and *Pseudomonas* sp. were only identified in Kiel Fjord samples.

Seawater samples from all three sampling sites were dominated by Proteobacteria and Bacteroidetes (Figure 1, Table S7). High abundance of Bacteroidetes contributed to the differences observed with ascidian microbiomes. Moreover, most of the OTUs abundant in the ascidian samples (Table 1 and Table S9) were much less abundant or absent from seawater samples. For example, higher abundances of Cyanobacteria in the Canadian population and of Firmicutes in Helgoland samples were not observed in the seawater references from the same sites (Figure 1, Tables S7–S9).

Consequently, the microbiome of *C. intestinalis* clearly correlates with sampling location (C, H, K) and sample type (G, T, W) and differed from the surrounding seawater.

### 3.2. Comparative Metabolomics of C. intestinalis Extracts

UPLC-QToF-MS/MS was used to mine the metabolome of organic extracts of the three different *C. intestinalis* populations. Ascidian samples were extracted at (i) population level (pooled samples of 13 g freeze-dried whole ascidian specimens per sampling site and replicate) to examine the ascidians’ global chemical profile at different locations and at (ii) individual level for comparing tunic and inner body tissues (see Table S2). Instead of the gut, we extracted the full inner body, since gut tissues did not yield enough extract for metabolomics.

**Figure 3:**
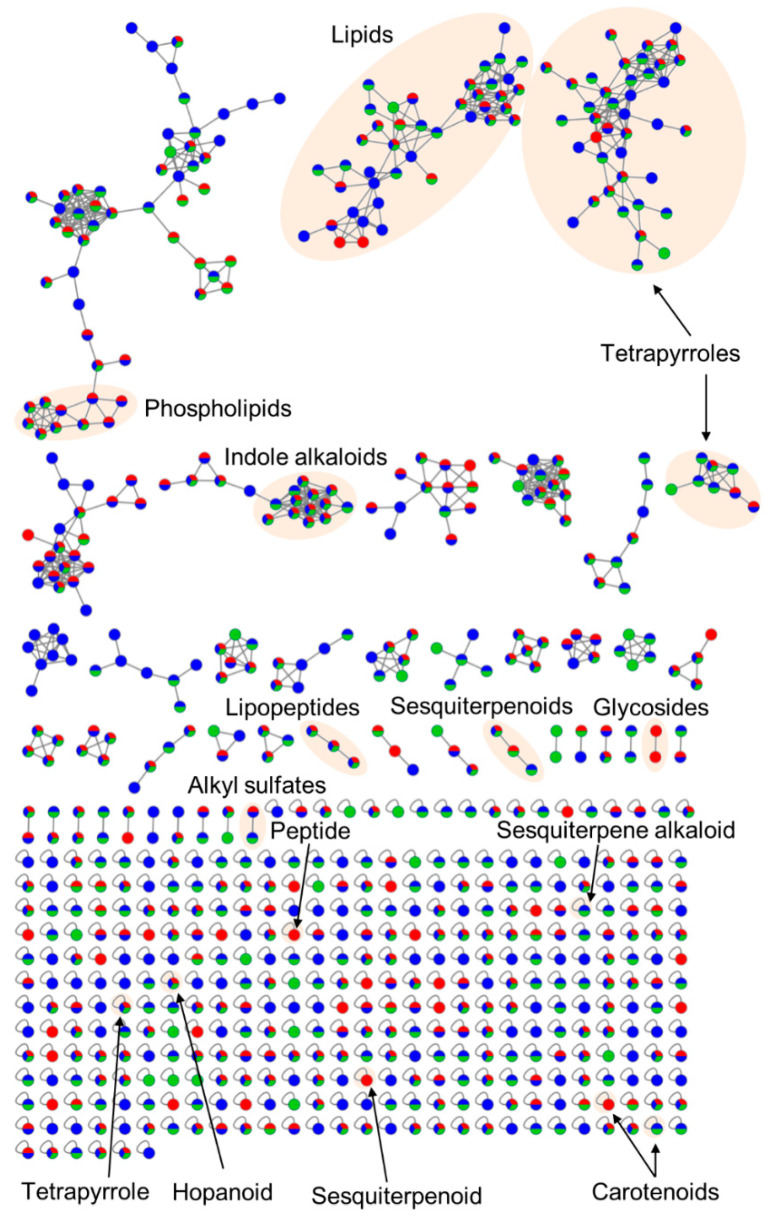
Global molecular network (MN) of three different *C. intestinalis* populations. The MN was constructed via the online platform GNPS [26] by using pre-filtered MS/MS-data of the population level metabolome study. Nodes are color-coded and reflect their sampling site: red: Canada, blue: Helgoland, green: Kiel. Putatively annotated clusters or nodes are highlighted.

Population extracts from Canadian samples yielded significantly lower quantity of extracts (0.3 g ± 0.01) compared to those from Helgoland (0.4 g ± 0.02) and Kiel Fjord (0.5 g ± 0.01; Figure S6; ANOVA: df: 2, F: 61.73, *p* < 0.0001, Tukey’s HSD for all comparisons *p* < 0.01). Manual peak picking yielded 121 abundant metabolites, which were annotated by manual and automated dereplication tools, i.e., DNP, MarinLit, GNPS [26], and ISDB-UNPD [27]. The MN, which contained 44 clusters, further aided the putative annotation and verification of known compounds (Figure 3). This combined approach led to the putative annotation of 41 compounds to known natural products (NPs) or chemical families, which translates into a high annotation rate of 34% (Figure S7, Table S11). Putatively annotated compounds showed a broad chemical diversity, such as alkaloids (**5**, **35**, **41**, **43**, **49**, **53**, **58**, **87**, **89**, **90**, **96**, **103**, **105**, **108**, **117**), lipids (**6**, **12**, **14**, **15**, **25**, **26**, **50**, **59**, **64**, **73**, **79**, **88**, **95**, **101**, **104**, **107**, **111**), peptides (**55**), polyketides (**75**, **100**), and terpenoids (**24**, **56**, **60**, **69**, **99**, **120**). The high abundance of lipids and terpenoids was confirmed by MN, since the three largest clusters were assigned to lipids and tetrapyrrole-type pigments (Figure 3). The majority of the putatively annotated NPs derived from ascidians or marine invertebrates (58%), but a considerably high portion of metabolites was of microbial origin (32%; Table S11). Nevertheless, two thirds of the detected peaks and many clusters in the MN remained unknown and may represent potentially new compounds.

With 1156 abundant peaks, the individual inner body and tunic metabolomes also exhibited a high chemical diversity (Figure S8). Several clusters in the MN were predominantly associated with either inner body or tunic and notably, 195 nodes were exclusively detected in tunic tissues, but only 98 in inner body samples. For instance, the tetrapyrrole purpurin 18 (**108**) was only present in the inner body of *C. intestinalis* and two carotenoids (**56**, **99**) were exclusively detected in its tunic (Table S11). Several metabolites were highly abundant in one tissue, e.g., several lipids were more often detected in either inner body (**12**, **14**, **50**) or tunic samples (**64**, **104**). Most of the putatively identified compounds, e.g., unsaturated fatty acids (**95**, **104**, **111**), indole alkaloids (**43**, **49**, **53**), and polyunsaturated amino alcohols (**12**, **14**, **64**), were detected in both tissues. Comparative metabolome analysis of individual ascidian samples proved tissue-specific metabolite profiles (R: 0.68, *p* = 0.0001; Figure S9, Table S12). Individuals from one sampling site showed variability and hence, geography-based chemotypes were much less apparent than in the population level analysis, i.e., samples did not cluster clearly with respect to the three sampling locations. Statistical testing confirmed little separation by sampling site (R: 0.23–0.45, *p* = 0.0025–0.0001; Table S12). Finally, individual tunic metabolite profiles were statistically tested for correlation with the respective individual microbial community compositions. In line with the lesser apparent geography-based clustering of individual metabolomes, no clear correlation was detected (R: 0.26, *p* = 0.002; Figure S10, Table S12).

**Figure 4:**
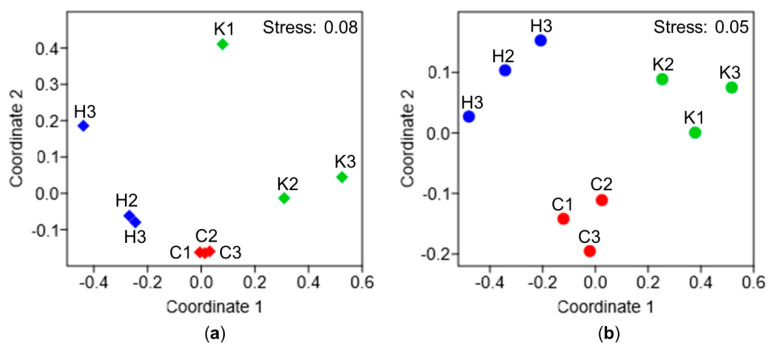
Comparative chemical profiling of geographically distinct *C. intestinalis* populations. Chemical profiling was performed with population level extracts. The 2D nMDS plot was constructed using (**a**) the full set of detected metabolites or (**b**) a restricted dataset containing only core metabolites detected in all samples. Similarity matrices were calculated with the Bray-Curtis similarity index based on the peak area. Sampling locations are color-coded: red: Canada (replicate C1–C3), blue: Helgoland (replicate H1–H3), green: Kiel (replicate K1–K3).

Comparison of the metabolomes at population level (pooled whole ascidian samples) showed distinct clustering relating to the three different sampling locations (Figure 4a). However, two replicates (H3, K1) deviated from their respective sample group. The main reason for these two outliers is the detection of a few peaks that were specifically enhanced or unique to these replicates (Table S11). When only core metabolites (i.e., detected in all samples) were compared, samples clearly clustered by their geographic origin (Figure 4b). Statistical comparison confirmed the observed clustering by sampling sites (R: 0.6–1), although ANOSIM results were insignificant (*p* > 0.05; Table S12).

**Figure 5:**
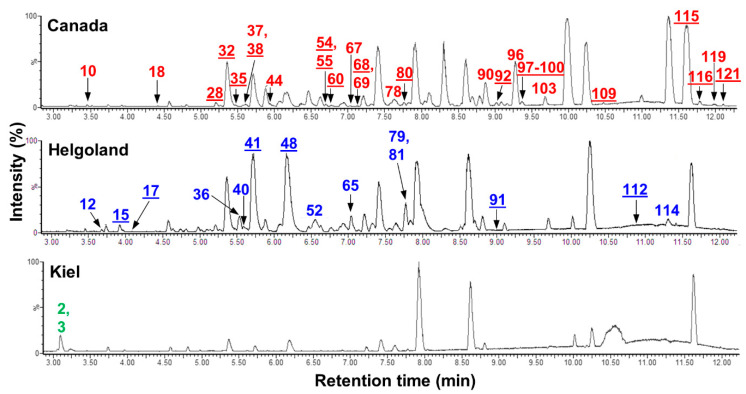
UPLC-MS chromatograms of *C. intestinalis* extracts from different sampling sites. For each sampling site, one representative extract was selected (Canada: C1, Helgoland: H2, Kiel: K2). Metabolites with enhanced or exclusive (underlined numbers) production in one of the three sampling locations are labelled with the respective peak number. Peak annotation is in accordance with Table S11 and annotated peaks were color-coded reflecting their sampling location (red: Canada, blue: Helgoland, green: Kiel).

In-depth chemical investigations of *C. intestinalis* from different locations revealed that most compounds (80%) were common to all population level extracts. However, 24 metabolites appeared to be unique to one sampling location while 19 metabolites had higher quantities in one sampling site (Table S11, Figure 5). Canadian and Helgoland samples were chemically the most diverse and contained the highest number of location-specific metabolites. Eighteen metabolites were exclusive and another eleven showed enhanced production (at least 10-fold higher abundance) in Canadian samples compared to extracts of Helgoland and Kiel specimens (Figure 5, Table S11). Only six compounds were exclusive to Helgoland population extracts and none were identified from Kiel samples. Eight compounds showed enhanced production in Helgoland samples, while only two compounds were enhanced in Kiel samples.

**Figure 6:**
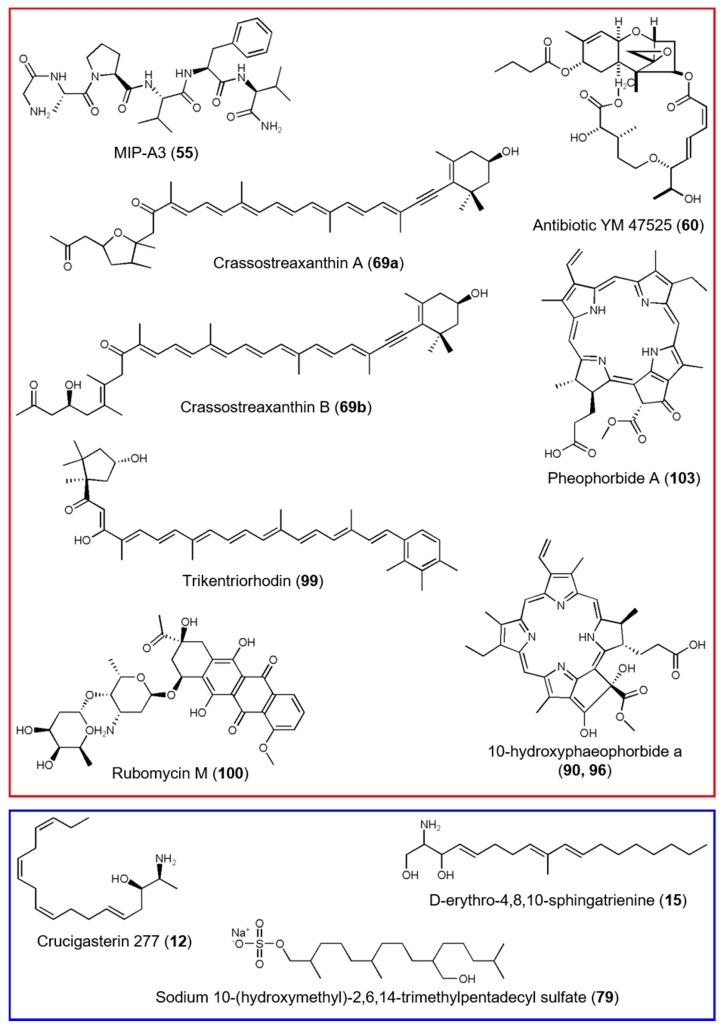
Metabolites with enhanced or exclusive production in *C. intestinalis* specimens from Canada (red) and Helgoland (blue). Peak annotation of putatively identified compounds from Canadian and Helgoland population extracts is in accordance with Table S11.

Of the 29 compounds that showed enhanced or exclusive production in Canadian extracts, nine were putatively identified as the peptide MIP-A3 (**55**), the sesquiterpenoid antibiotic YM 47525 (**60**), and the anthracycline polyketide rubomycin M (**100**), plus six pigments belonging to carotenoid (**69**, **99**) and tetrapyrrole (**35**, **90**, **96**, **103**) classes (Figure 6). Four metabolites specific to Helgoland samples were putatively annotated to the sphingolipids crucigasterin 277 (**12**) and d-erythro-4,8,10-sphingatrienine (**15**), the alkyl sulfate sodium 10-(hydroxymethyl)-2,6,14-trimethylpentadecyl sulfate (**79**), and the chemical family of tetrapyrroles (**41**). The two compounds with enhanced production in *C. intestinalis* from Kiel Fjord could not be linked to any known NP and remain therefore unknown.

The crude extracts were visually different, i.e., the Canadian *C. intestinalis* extracts had a strong green color, whereas Helgoland and Kiel Fjord samples appeared orange (Figure S11). This color difference may (partly) be explained by the presence of four Canada-specific tetrapyrrole-type pigments (**35**, **90**, **96**, **103**; Figure 5 and Figure 6, Table S11).

## 4. Discussion

In order to gain first insights on potential factors contributing to the invasiveness of *C. intestinalis,* we comparatively profiled the microbiome and metabolome of different tissues of native and invasive specimens. The overall bacterial diversity of the tunic of specimens from all three locations was comparable to that of *C. robusta*, but higher than previously reported for *C. intestinalis* and *C. savignyi* [57,58] (Table S4). Dominance of Alphaproteobacteria and Bacteroidetes in the tunic (Figure 1, Tables S7 and S8) is in line with previous results for *C. intestinalis* from the North Atlantic coast [57]. Additionally, the composition of the ascidian’s gut microbiome is largely consistent with a previous study [59], although we found higher abundances of Actinobacteria, Tenericutes, and Verrucomicrobia. Our results showed a higher alpha diversity for specimens from the invasive population (Canada) compared to the native populations from Kiel and Helgoland (Tables S4 and S5). Further studies including several invasive populations are necessary to confirm this finding and shed light into the impact of invasiveness vs. population variation on alpha diversity.

Species-specific microbial assemblages are well-known for ascidians [60,61,62], including *C. intestinalis* [58]. Accordingly, ascidian microbiomes analyzed in this study differed significantly from the surrounding seawater (Figure 1 and Figure 2, Tables S4, S6–S9). For instance, while some bacterial taxa such as Cyanobacteria (Canada) and Firmicutes (Helgoland guts) were highly abundant in the ascidian microbiome, these taxa showed extremely low abundance in the ambient seawater samples. We detected several abundant ascidian-specific OTUs accounting for 20% of the total sequence reads (OTUs 1–3, 7, 10, 16), which were previously reported for *C. intestinalis* from e.g., the NW Atlantic US-coast [57,59] (Table 1, Table S9 and S10). This corroborates that some abundant bacteria are stably associated with *C. intestinalis* across different geographic scales and seasons [58,59]. We further demonstrated tissue-specific associations of bacteria (Figure 2 and Figure S4, Tables S6–S9), which represents to our knowledge the first comparative study of the gut and tunic microbiome. Location-specific microbial patterns were more prevalent than tissue-specificity contrasting a previous study showing a geographically conserved microbiome of *Ciona* spp. [58]. This may be explained by different sampling strategies; we sampled from different regions (Atlantic Ocean, North and Baltic Seas), while the previous study had comparably low levels of geographic separation (<300 km distance; [58]). Geography-based microbial variation is a well-known phenomenon that was previously described for e.g., the invasive ascidian *Herdmania momus* [61]. Site-specific microbial patterns can be attributed to different environmental conditions, such as salinity, i.e., oceanic (Helgoland, SH, Canada) versus brackish (Kiel), and different levels of anthropogenic input (high: Kiel, Canada; low: offshore-island Helgoland). For example, the high abundance of *Arcobacter* sp. (Epsilonproteobacterium; Tables S8 and S9) in Kiel samples may point to fecal pollution at this location, since *Arcobacter* sp. correlates with fecal pollution but not salinity [63]. Firmicutes dominated the gut of Helgoland ascidians, including *Clostridium* sp. (Figure 1, Table 1, Tables S7–S9) that is known as a common gut symbiont of marine fish supplying them with e.g., fatty acids [64]. Nevertheless, reasons for this specific association of Firmicutes with the gut of Helgoland *C. intestinalis* remain obscure.

In addition to environmental factors shaping the microbiome of the three different populations, haplotype-diversity of the host may also have impacted the microbial diversity of *C. intestinalis*. A recent study on the microbiome of colonial ascidian *Clavelina oblonga* revealed lower haplotype-diversity in the invasive populations, suggesting that lower genetic diversity correlates with the decreased microbial diversity detected in the invasive specimens [65]. However, the investigation of the impact of genetic diversity on microbial diversity is beyond the scope of the present study.

To investigate the contribution of the microbiome to the overall fitness of *C. intestinalis*, we focused on bacterial taxa conveying potential beneficial effects for *C. intestinalis* (Tables S7–S10). Cyanobacteria, which were abundant in the gut and tunic (only Canadian specimens) of *C. intestinalis*, are known to produce a variety of toxins against e.g., marine invertebrates and fishes [66]. For instance, cytotoxic and antibacterial compounds were already isolated from *Leptolyngbya* spp. [67,68], a cyanobacterial genus detected only in Canadian *C. intestinalis* samples. The Alphaproteobacteria *Roseobacter* sp. (mainly Canada), *Ruegeria* sp. (mainly Canada and Helgoland), and *Kiloniella* sp. (mainly Helgoland and Kiel) and the Cyanobacterium *Synechococcus* sp. (all sites) are known producers of bioactive compounds such as antimicrobial and antifouling agents [69,70,71,72]. Moreover, several bacteria such as *Arcobacter* sp. (mainly Kiel), *Roseobacter* sp. (mainly Canada), and *Ruegeria* sp. (mainly Canada and Helgoland) play a role in nitrogen cycling [65,72] and hence, may thrive in eutrophic habitats such as PEI and Kiel Fjord [60,61]. Additionally, symbiosis with heavy metal resistant bacteria (known from some Alteromonadales and *Vibrio* spp., observed at all three sampling sites) may be beneficial for *C. intestinalis*, especially in marine areas with high anthropogenic activity [60,61]. Specific associations of bacterial taxa with capacity to provide ecologically relevant functions (e.g., chemical defense, heavy metal resistance) may enhance the overall performance of *C. intestinalis*. Further investigations analyzing several invasive populations are needed to verify the role of these microbes, in particular those specific to Canadian ascidians, for the global expansion of *C. intestinalis*.

Previous studies suggested that both symbiont recruitment strategies, vertical and horizontal transmission, play crucial, complementary roles for survival, but also establishment of invasive ascidians in new ecosystems [25,60,61,73]. Vertical symbiont transmission (intergenerational transfer of core microbes) ensures stability of core microbiota and their functions in host health, but is considered to lower the adaptability of the macrobiont [25,61]. Horizontal transmission (active recruitment of beneficial microorganisms from the surrounding seawater) plays an important role for invasive species by enabling rapid acclimation to the new habitat [25,61,74]. In this study, we also observed a combination of location-independent core and site-specific microbial signatures in all three sampled populations, i.e., several abundant OTUs were detected either across broad geographical ranges (in this and previous studies; core microbes) or were exclusive to one sampling site (site-specific microbes). Hence, these results corroborate previous findings indicating a combination of vertical and horizontal symbiont transmission [25,60,61,73].

The integrated metabolomics approach combining automated and manual dereplication tools clearly outperformed classical dereplication techniques [75] by significantly increasing annotation rates (this study: combined 34%, max. 12% for single methods, Table S11). Similar to previous studies on ascidians [62,76], we detected a high abundance of lipids (41%; Figure 3 and Figure S7, Table S11). Alkaloids were also abundant (37%), which was expected since ascidians are prolific producers of alkaloids [77,78]. Being the sister group of vertebrates, ascidians have a rich repertoire of secondary metabolites [78,79] and with 19 putatively annotated molecular families, *C. intestinalis* showed a diverse metabolome. Many putatively annotated metabolites were previously isolated from other marine invertebrates indicating a microbial/microalgal origin, as reported for 10-hydroxyphaeophorbide a (**90**, **96**) [80]. We putatively identified a comparably high share of microbial metabolites (32%), providing support for higher abundance of microbial metabolites in ascidians than previously estimated [78,81]. Furthermore, we detected the bacterial producers (e.g., *Moorea* sp. and *Bacillus* sp.) of several putatively identified compounds (**26**, **43**, **59**, **88**, **117**) in the tunicate microbiome. However, we did not observe a clear correlation between the individual tunic microbiomes and metabolomes (Figure S10), which may be due to multiple drivers, such as diet, age, stress, abiotic environmental parameters, phenotypic plasticity, and inter-individual genetic variations, influencing the microbial community and metabolite production on individual level [82,83,84].

Remarkable location- and tissue-specific patterns were also observed in the metabolome of individual and population samples (Figure 4 and Figure 5, Figure S9, Tables S11 and S12). Location-specific metabolites are commonly reported, also from ascidians [62]. The individual metabolomes revealed lesser pronounced site-specific signatures possibly due to high inter-individual variability [82,85]. Moreover, the tissue type (inner body vs. tunic) had a significant impact on individual metabolomes. This is in line with a study reporting differential lipid composition of inner body and tunic extracts of *C. intestinalis* [76]. Notably, the native population from Kiel Fjord showed the lowest chemical diversity. In the brackish Kiel Fjord salinities go down to approximately 12 psu (average salinity 18 psu), which is at the lower tolerance limit of *C. intestinalis* [4]. Salinity can influence the metabolite production of e.g., the clam *Ruditapes philippinarum* [86] and therefore, the comparably low salinity in Kiel Fjord may have contributed to the lower chemical diversity.

Similar to the microbiome, we inspected putatively annotated compounds with regard to their known bioactivities. Several putatively annotated metabolites are reportedly cytotoxic (**43**, **87**, **89**, **103**, **105**) or have antimicrobial activities (**14**, **60**, **87**, **89**, **100**). Notably, while most bioactive compounds were present in all three populations (**14**, **43**, **87**, **89**, **103**, **105**), two metabolites (**60**, **100**) were only detected in the invasive population from Canada. The putatively identified antibiotic YM 47525 (**60**) has been shown to be fungicidal against *Candida albicans* [87], thus may provide *C. intestinalis* with protection against pathogenic fungi. Likewise, the anthracycline polyketide rubomycin M (**100**) that was putatively annotated in the Canadian population is a potent antibiotic [88]. The detected bioactive secondary metabolites may contribute to the chemical defense of *C. intestinalis*, a finding previously reported from various other ascidians [62,83].

Evidence from the terrestrial biosphere suggests that the invasion success of plants is promoted via a richer and more specific chemical repertoire [89,90]. This may also account for *C. intestinalis*, since we observed higher chemical diversity in invasive specimens (Figure 5 and Figure 6, Table S11). Furthermore, we observed significantly lower extract yields with invasive *C. intestinalis* specimens (Figure S6). As outlined above, this phenomenon, a shifted metabolism towards growth and reproduction, is known as the “evolution of increased competitive ability hypothesis” [8], which may be one explanation for the lower quantity of the Canadian ascidian extracts. These two interesting findings require further studies on additional invasive populations to identify the impact of invasiveness, population variation, and environmental variation.

In this study, we simultaneously examined for the first time the metabolome and microbiome of native and invasive specimens of *C. intestinalis*. Both the geographical location and the tissue type (either gut or tunic) significantly impacted the microbial community composition and metabolite profiles of different *C. intestinalis* populations. Stable core OTUs and tissue- or location-specific bacterial taxa and metabolites suggest a high degree of flexibility when adapting to new environmental conditions. While additional sampling is needed to verify the role of microbes and metabolites during invasion, our results give first evidence that invasive *C. intestinalis* contain a richer microbiome and metabolome that may enhance its adaptive capacity in the new environment. Several ascidian-associated microbes have reported bioactivities (e.g., antimicrobial) or other ecologically relevant functions (e.g., nitrogen metabolization, antifouling), potentially contributing to the health and fitness of *C. intestinalis*. Some putatively annotated metabolites may provide beneficial bioactivities (e.g., antimicrobial) supporting the ascidian’s chemical defense. Hence, beneficial microbiota and chemical weapons may be relevant factors in addition to other reported characteristics (e.g., broad tolerance of abiotic environmental parameters, fast accumulation of biomass, high fertility) regarding the global expansion of *Ciona* species. Additional studies on several invasive and native populations across broad geographical scales will be necessary to understand the contribution of the microbiota and metabolome to its global invasion success.

## Figures and Tables

**Table 1:** Frequent bacterial OTUs (≥1%) with significantly different abundance across nine different sample groups (sample type and sampling location). The predominant sample type and (if applicable) sampling location (in brackets) in this study are indicated for each OTU. OTUs were classified with BLAST down to the lowest possible taxonomic rank and are given with the accession number and isolation source. Relative abundances, statistics, and full BLAST results are given in Tables S9 and S10. Ubc: Uncultured bacterium clone.

OTU	Lowest Classification (BLAST)	Accession Number (BLAST)	Isolation Source According To BLAST	Dominant Sample Type (Location) This Study
OTU1	*Kordiimonas* sp.	KF494349.1	*Ciona intestinalis* (tunic)	Tunic
OTU2	Rhizobiales	MN006421.1	Various, e.g., *Ciona intestinalis* (tunic)	Tunic
OTU3	*Arenibacter* sp.	KF494352.1	*Ciona intestinalis* (tunic)	Tunic
OTU4	*Pseudomonas* sp.	MH244157.1	Sediment	Gut (Kiel)
OTU6	*Synechococcus* sp.	MH358353.1	Marine environment	Gut
OTU7	Uncultured alphaproteobacterium_1-21	FJ659126.1	Ascidian (*Aplidium conicum*; tunic)	Tunic
OTU8	*Synechococcus* sp.	KU867940.1	Seawater	Gut
OTU10	Ubc Woods-Hole_a5143	KF798938.1	*Ciona intestinalis* (gut)	Gut
OTU11	Rhodobacteraceae	KU173743.1	Seawater	Seawater
OTU13	*Bifidobacterium dentium*	LR134349.1	Human	Gut (Helgoland)
OTU15	*Roseobacter* sp.	MK224709.1	Red algae	Gut (Canada)
OTU16	Ubc Woods-Hole_a5449	KF799049.1	*Ciona intestinalis* (gut)	Gut
OTU17	*Litoreibacter* sp.	KJ513684.1	Seawater	Several

## Supplementary Materials

The following are available online at https://www.mdpi.com/2076-2607/8/12/2022/s1, Figure S1: Genotyping of *C. intestinalis* with the mitochondrial marker gene COX3-ND1, Figure S2: Influence of the quality filtering steps on the total number of observed read pairs from amplicon sequencing, Figure S3: Rarefaction curves of OTU abundances for *C. intestinalis* and seawater samples, Figure S4: Multivariate ordination plots of the bacterial community associated with *C. intestinalis*, Figure S5: Across sample type and geographic origin comparison of the *C. intestinalis* associated microbiome, Figure S6: Extraction yields of crude extracts from population level extractions, Figure S7: Chemical structures of putatively identified compounds in crude extracts of *C. intestinalis* by UPLC-MS/MS analysis, Figure S8: Molecular network (MN) of individual *C. intestinalis* metabolomes, Figure S9: Multivariate ordination plots of UPLC-MS profiles of *C. intestinalis* extracts, Figure S10: Statistical correlation of individual tunic microbiomes and metabolomes, Figure S11: Solvent extracts of different *C. intestinalis* samples, Table S1: Metadata for microbiome samples analyzed in this study, Table S2: Metadata for metabolome samples analyzed in this study, Table S3: Parameters of the individual extractions, Table S4: Alpha diversity measures of amplicon sequences, Table S5: Tukey’s HSD test comparing observed (OTU count), estimated (Chao1) OTUs, and phylogenetic diversity (PD) detected in ascidian samples at three different sampling sites, Table S6: ANOSIM comparison of amplicon sequencing results, Table S7: Significantly different abundant bacterial phyla, Table S8: Significantly different abundant bacterial classes, families and genera, Table S9: Significantly different abundant OTUs, Table S10: Classification of abundant OTUs detected in this study, Table S11: Putative annotation of metabolites detected in *C. intestinalis* bulk extracts (population level), Table S12: ANOSIM comparison of UPLC-MS/MS profiles of *C. intestinalis* extracts.
