# Supplementary material for: Comparative Microbiome and Metabolome Analyses of the Marine Tunicate Ciona intestinalis from Native and Invaded Habitats
Source: Microorganisms. 2020 Dec 17;8(12):2022. doi: 10.3390/microorganisms8122022 (PMC7767289; doi:10.3390/microorganisms8122022)
Supplement: Supplementary file 1 [file microorganisms-08-02022-s001.pdf]

Supplementary Information

Comparative Microbiome and Metabolome Analyses of the Marine Tunicate *Ciona intestinalis* from Native and Invaded Habitats

Caroline Utermann [1], Martina Blümel [1], Kathrin Busch [2], Larissa Buedenbender [1], Yaping Lin [3,4], Bradley A. Haltli [5], Russell G. Kerr [5], Elizabeta Briski [3], Ute Hentschel [2,6], Deniz Tasdemir [1,6]*

[1] GEOMAR Centre for Marine Biotechnology (GEOMAR-Biotech), Research Unit Marine Natural Products Chemistry, GEOMAR Helmholtz Centre for Ocean Research Kiel, Am Kiel-Kanal 44, 24106 Kiel, Germany

[2] Research Unit Marine Symbioses, GEOMAR Helmholtz Centre for Ocean Research Kiel, Duesternbrooker Weg 20, 24105 Kiel, Germany

[3] Research Group Invasion Ecology, Research Unit Experimental Ecology, GEOMAR Helmholtz Centre for Ocean Research Kiel, Duesternbrooker Weg 20, 24105 Kiel, Germany

[4] Chinese Academy of Sciences, Research Center for Eco-Environmental Sciences, 18 Shuangqing Rd., Haidian District, Beijing, 100085, China

[5] Department of Chemistry, University of Prince Edward Island, 550 University Avenue, Charlottetown, PE C1A 4P3, Canada

[6] Faculty of Mathematics and Natural Sciences, Kiel University, Christian-Albrechts-Platz 4, Kiel 24118, Germany

* Corresponding author: Deniz Tasdemir (dtasdemir@geomar.de)

This document includes:

Supplementary Figures S1-S11

Figure S1. Genotyping of *C. intestinalis* with the mitochondrial marker gene COX3-ND1.
Figure S2. Influence of the quality filtering steps on the total number of observed read pairs from amplicon sequencing.
Figure S3. Rarefaction curves of OTU abundances for *C. intestinalis* and seawater samples.
Figure S4. Multivariate ordination plots of the bacterial community associated with *C. intestinalis*.
Figure S5. Across sample type and geographic origin comparison of the *C. intestinalis* associated microbiome.
Figure S6. Extraction yields of crude extracts from population level extractions.
Figure S7. Chemical structures of putatively identified compounds in crude extracts of *C. intestinalis* by UPLC-MS/MS analysis.
Figure S8. Molecular network (MN) of individual *C. intestinalis* metabolomes.
Figure S9. Multivariate ordination plots of UPLC-MS profiles of *C. intestinalis* extracts.
Figure S10. Statistical correlation of individual tunic microbiomes and metabolomes.
Figure S11. Solvent extracts of different *C. intestinalis* samples.

Supplementary Tables S1-S12

Table S1. Metadata for microbiome samples analyzed in this study.
Table S2. Metadata for metabolome samples analyzed in this study.
Table S3. Parameters of the individual extractions.
Table S4. Alpha diversity measures of amplicon sequences.
Table S5. Tukey's HSD test comparing observed (OTU count), estimated (Chao1) OTUs, and phylogenetic diversity (PD) detected in ascidian samples at three different sampling sites.
Table S6. ANOSIM comparison of amplicon sequencing results.
Table S7. Significantly different abundant bacterial phyla.
Table S8. Significantly different abundant bacterial classes, families and genera.
Table S9. Significantly different abundant OTUs.
Table S10. Classification of abundant OTUs detected in this study.
Table S11. Putative annotation of metabolites detected in *C. intestinalis* bulk extracts (population level).
Table S12. ANOSIM comparison of UPLC-MS/MS profiles of *C. intestinalis* extracts.

Supplementary References 1-38

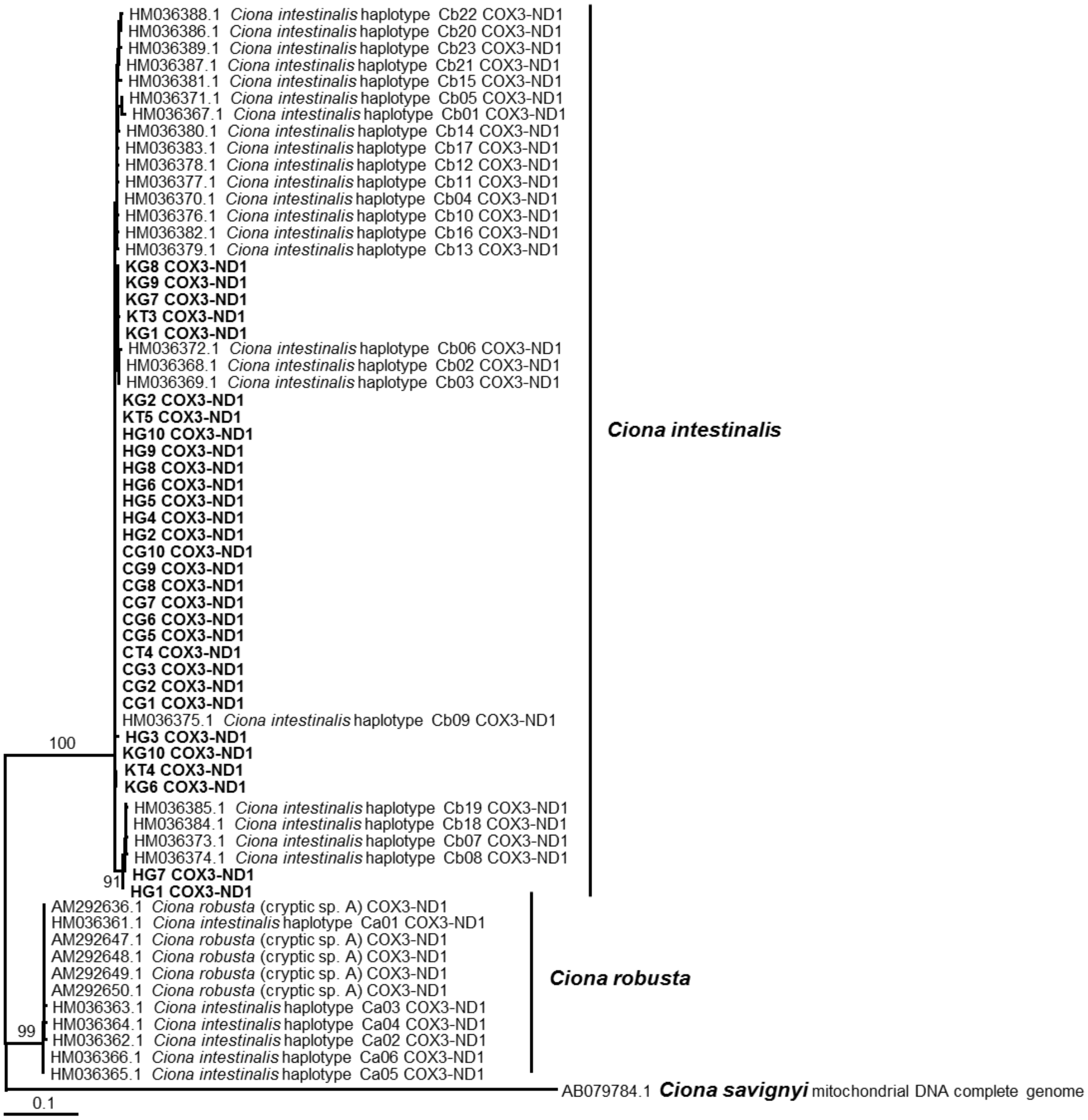

Figure S1. Genotyping of *C. intestinalis* with the mitochondrial marker gene COX3-ND1. A maximum likelihood tree was constructed in MEGA7. Genotyped individuals (n = 30) are in bold. Sampling sites and types are abbreviated as follows: C = Canada, H = Helgoland, K = Kiel; G = gut, T = tunic.

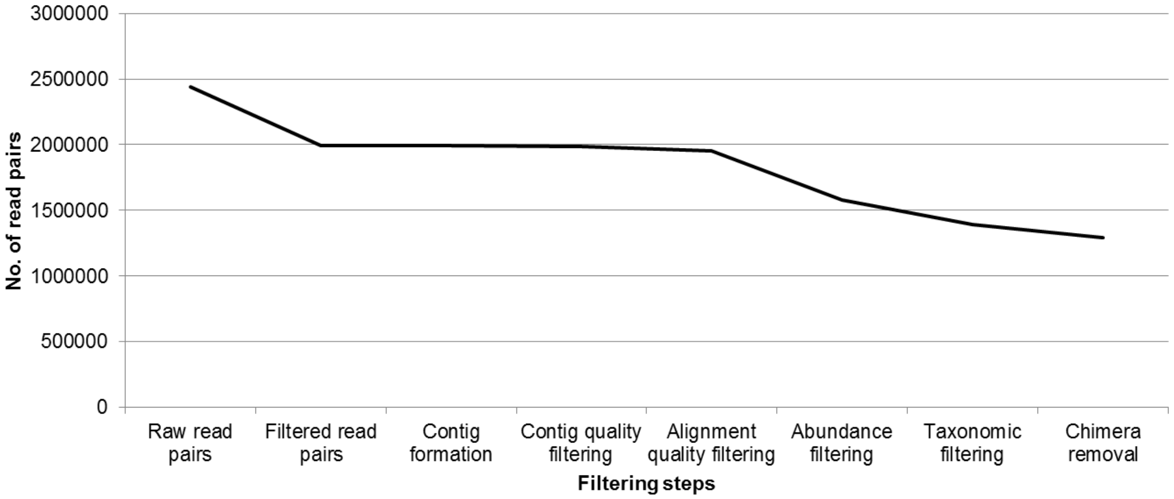

Figure S2. Influence of the quality filtering steps on the total number of observed read pairs from amplicon sequencing. Amplicon sequences were quality filtered in seven steps. The corresponding number of read pairs remaining after each filtering step is shown.

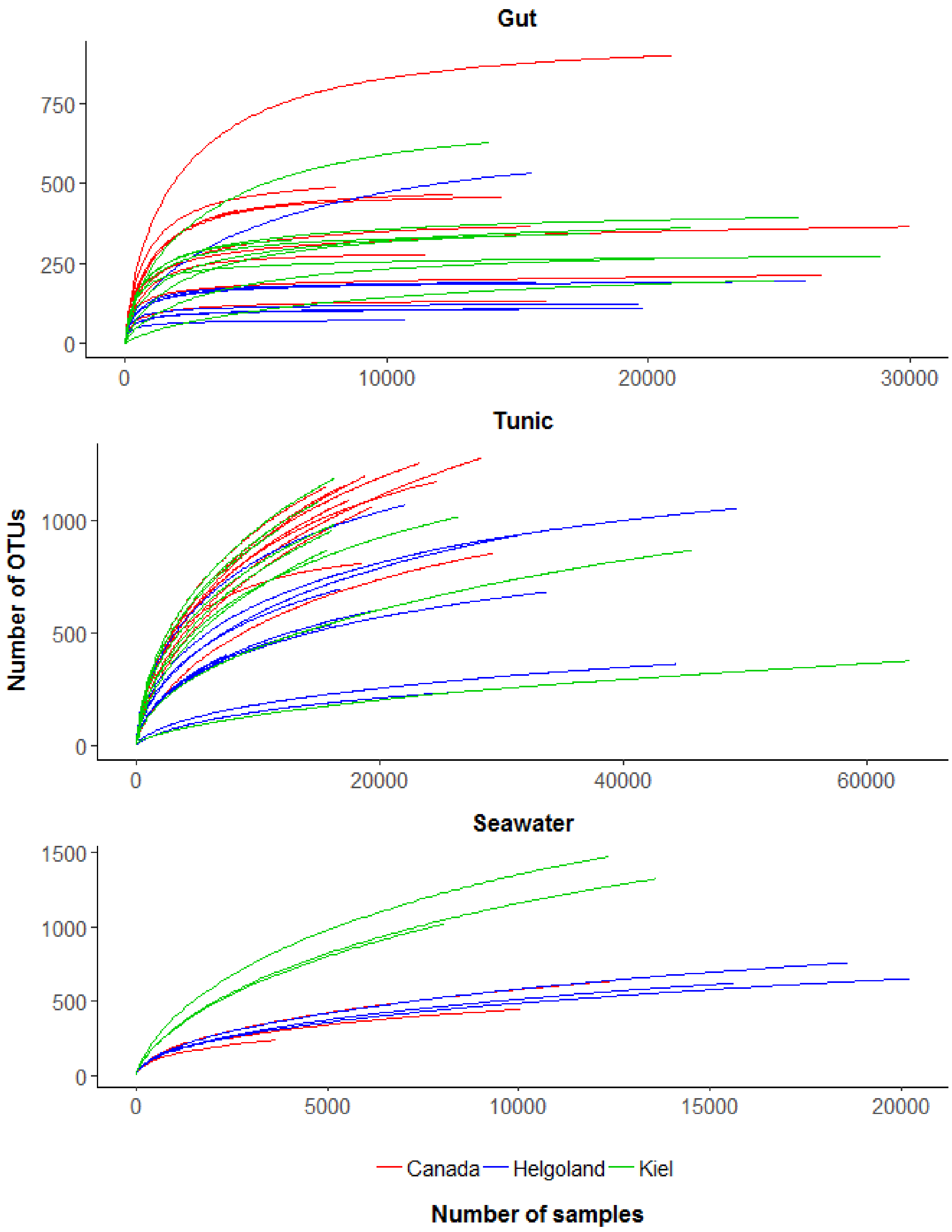

Figure S3. Rarefaction curves of OTU abundances for *C. intestinalis* and seawater samples. The number of sequences is plotted against the number of detected OTUs. Sampling sites are color coded: red = Canada, blue = Helgoland, green = Kiel Fjord. Top: gut, middle: tunic, bottom: seawater reference samples.

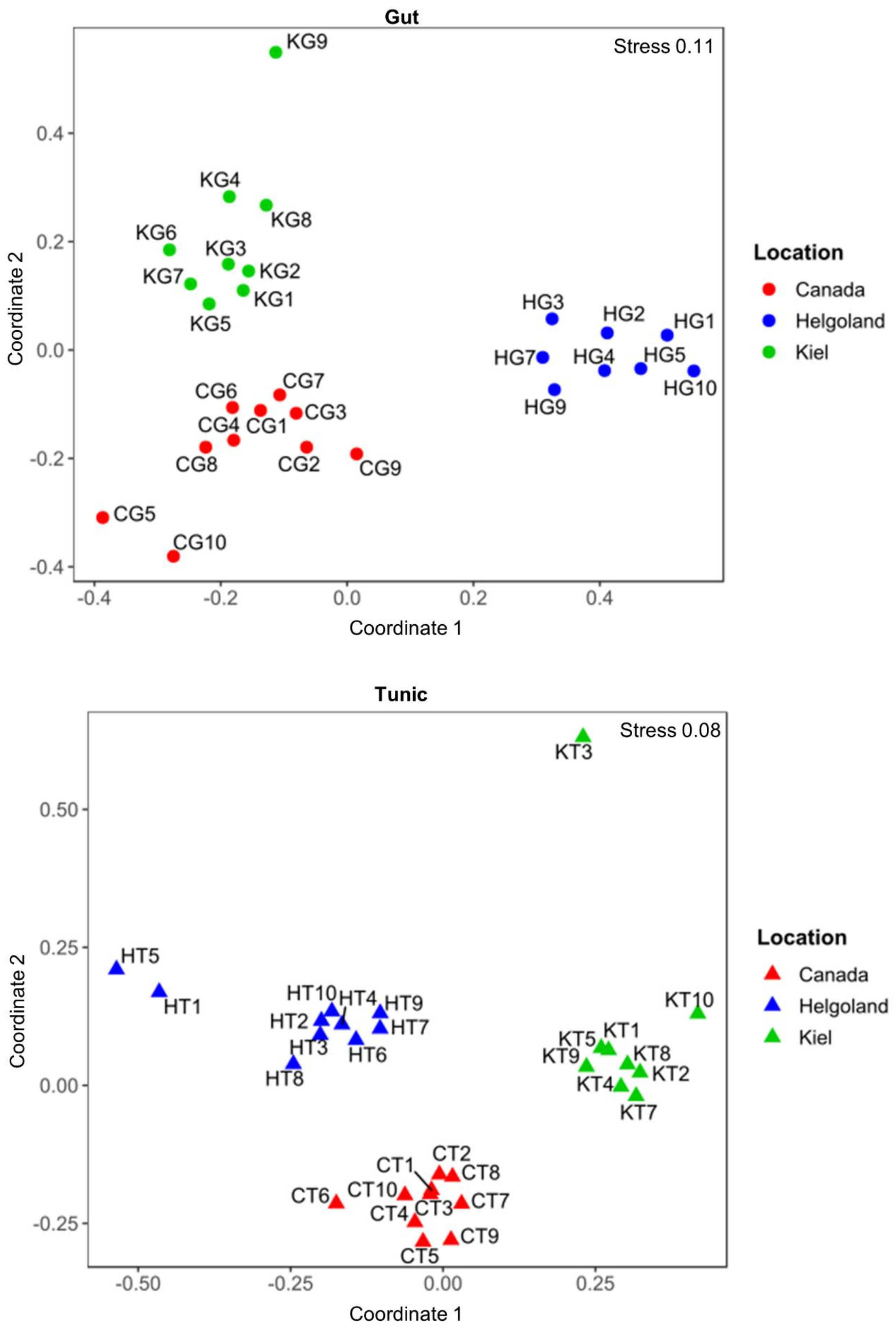

Figure S4. Multivariate ordination plots of the bacterial community associated with *C. intestinalis*. NMDS plots are based on Bray-Curtis similarity. Sampling sites and types are abbreviated as follows: C = Canada, H = Helgoland, K = Kiel; G = gut, T = tunic.

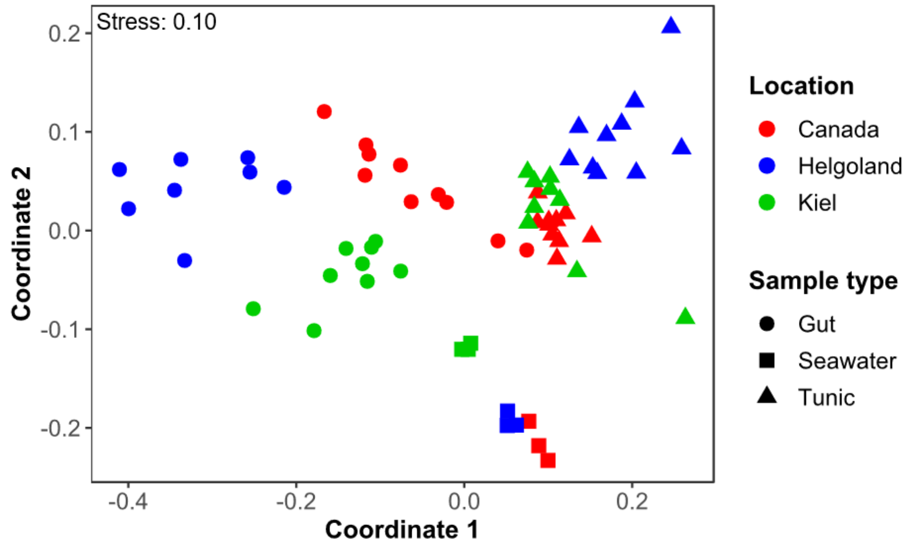

Figure S5. Across sample type and geographic origin comparison of the *C. intestinalis* associated microbiome. The 2D nMDS plot was calculated using the full set of detected OTUs (5211) and is based on weighted UniFrac distances.

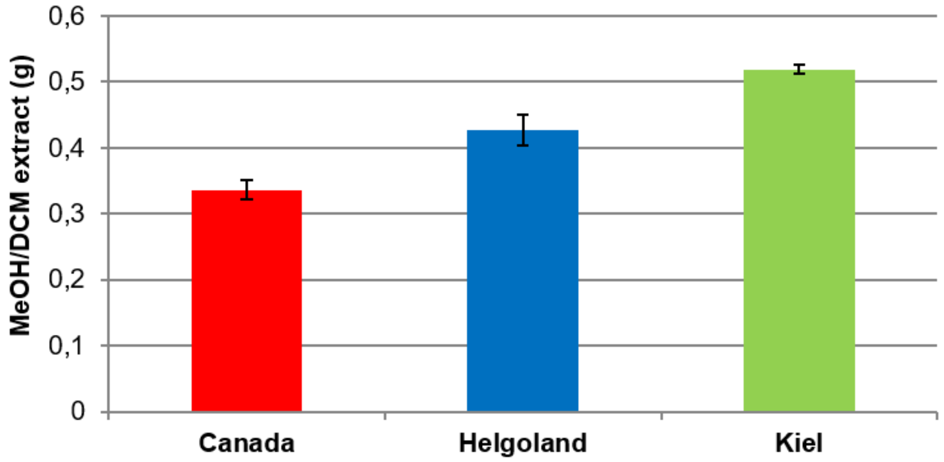

Figure S6. Extraction yields of crude extracts from population level extractions. Whole body bulk samples consisting of each 13 g of dry powder were extracted (n = 3 for each sampling site). Yields are given as average values with standard deviation in g.

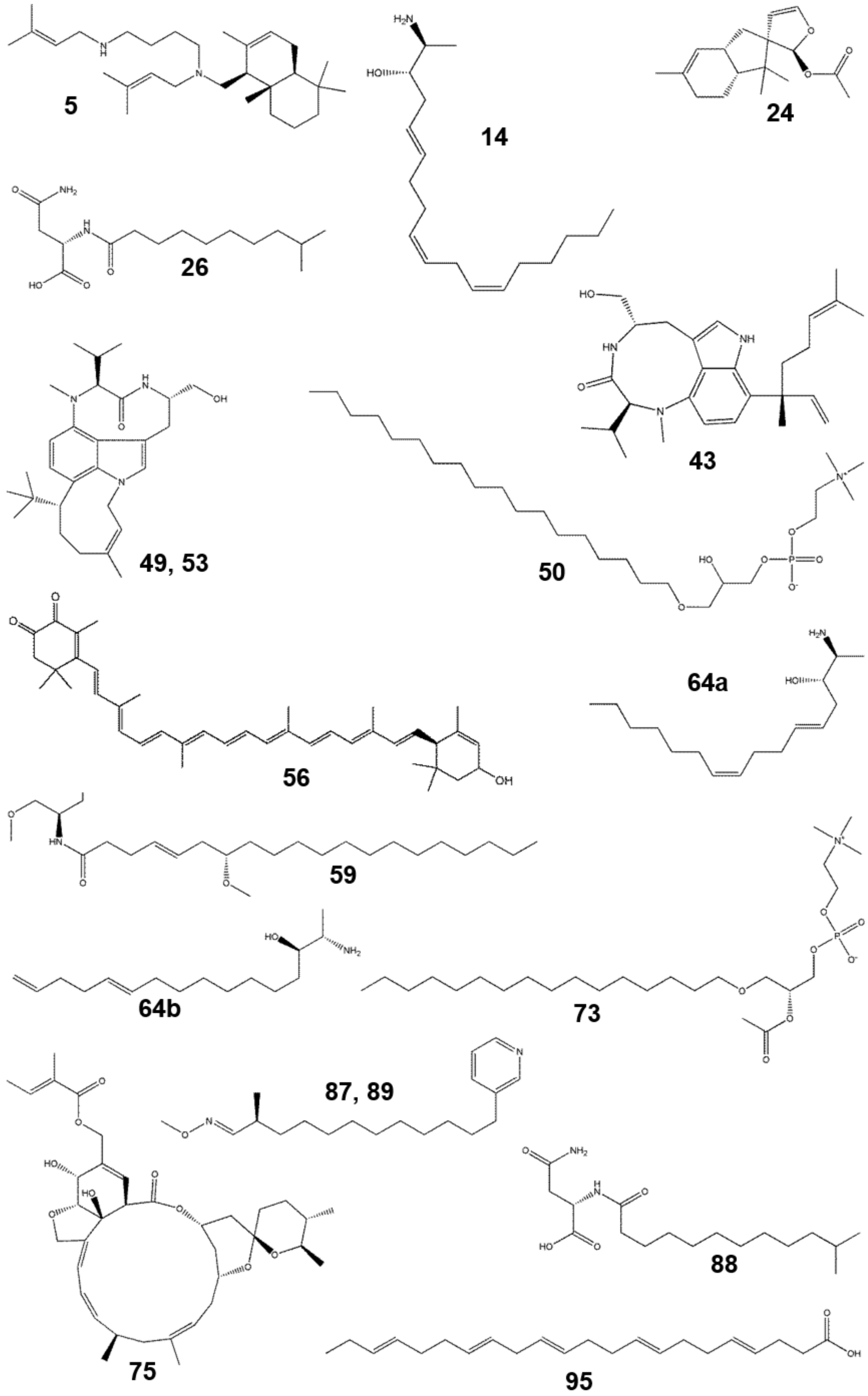

Figure S7. Chemical structures of putatively identified compounds in crude extracts of *C. intestinalis* by UPLC-MS/MS analysis. Structures are given with their respective peak number (see Table S11). The following compounds are shown in Figure 6 in the original publication: **12, 15, 55, 60, 69, 79, 90, 96, 99, 100, 103**.

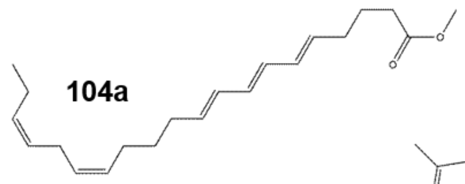

104a

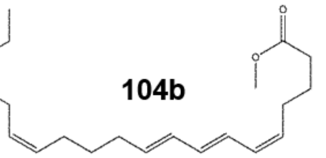

104b

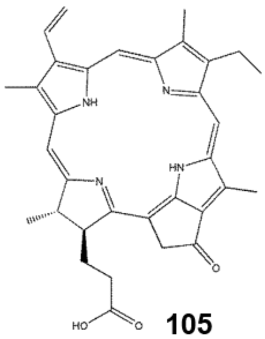

105

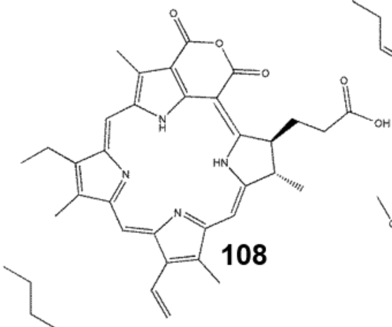

108

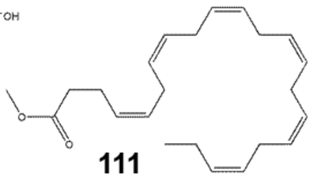

111

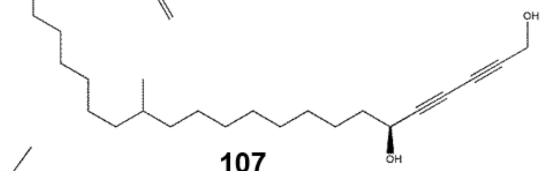

107

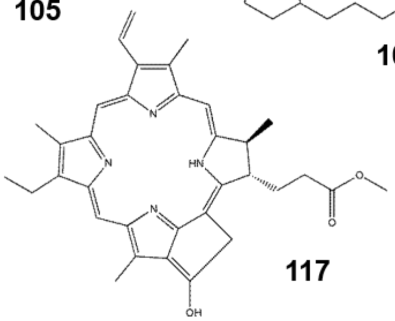

117

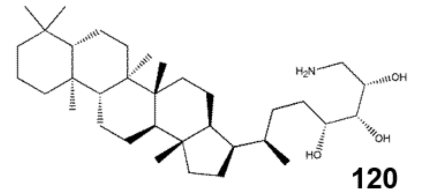

120

Figure S7. (continued)

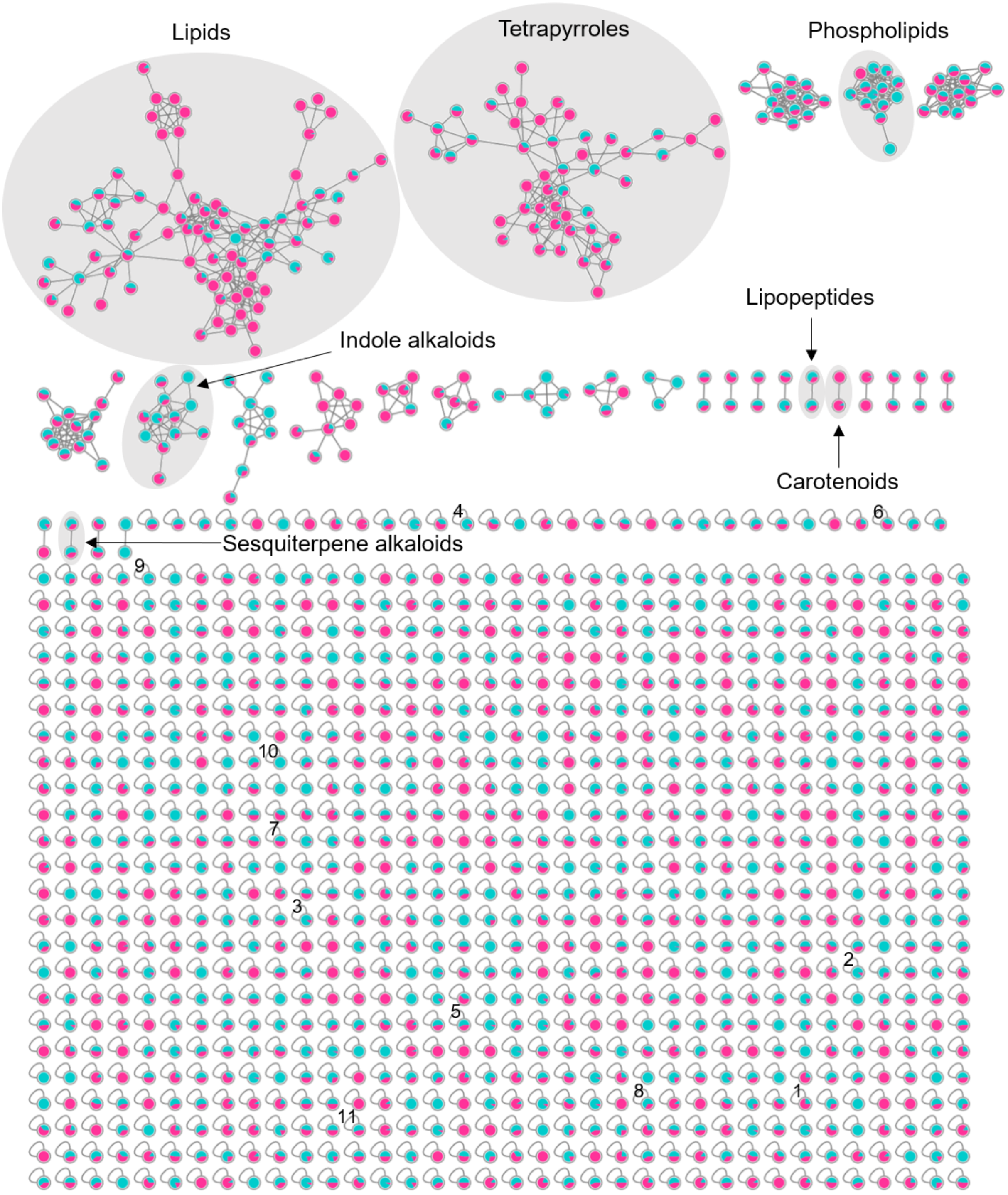

Figure S8. Molecular network (MN) of individual *C. intestinalis* metabolomes. The MN was constructed via the online platform GNPS [1] by using pre-filtered MS/MS-data of individual level extracts from inner body and tunic (ions must occur in ≥5 replicates). Nodes are color-coded and reflect the respective tissue: pink = tunic, cyan = inner body. Proportions are given by the number of replicates containing a respective node. Single nodes are numbered and were putatively annotated to the following chemical families: 1, 3, 4 = polyunsaturated amino alcohols; 2 = sesquiterpenoid; 5 = lipoamide; 6 = alkylpyridine; 7 = unsaturated fatty acid; 8 = acetylenic alcohol; 9 = hopanoid; 10 = tetrapyrrole; 11 = linear peptide. Putative annotations are in accordance with population metabolomes (Table S11).

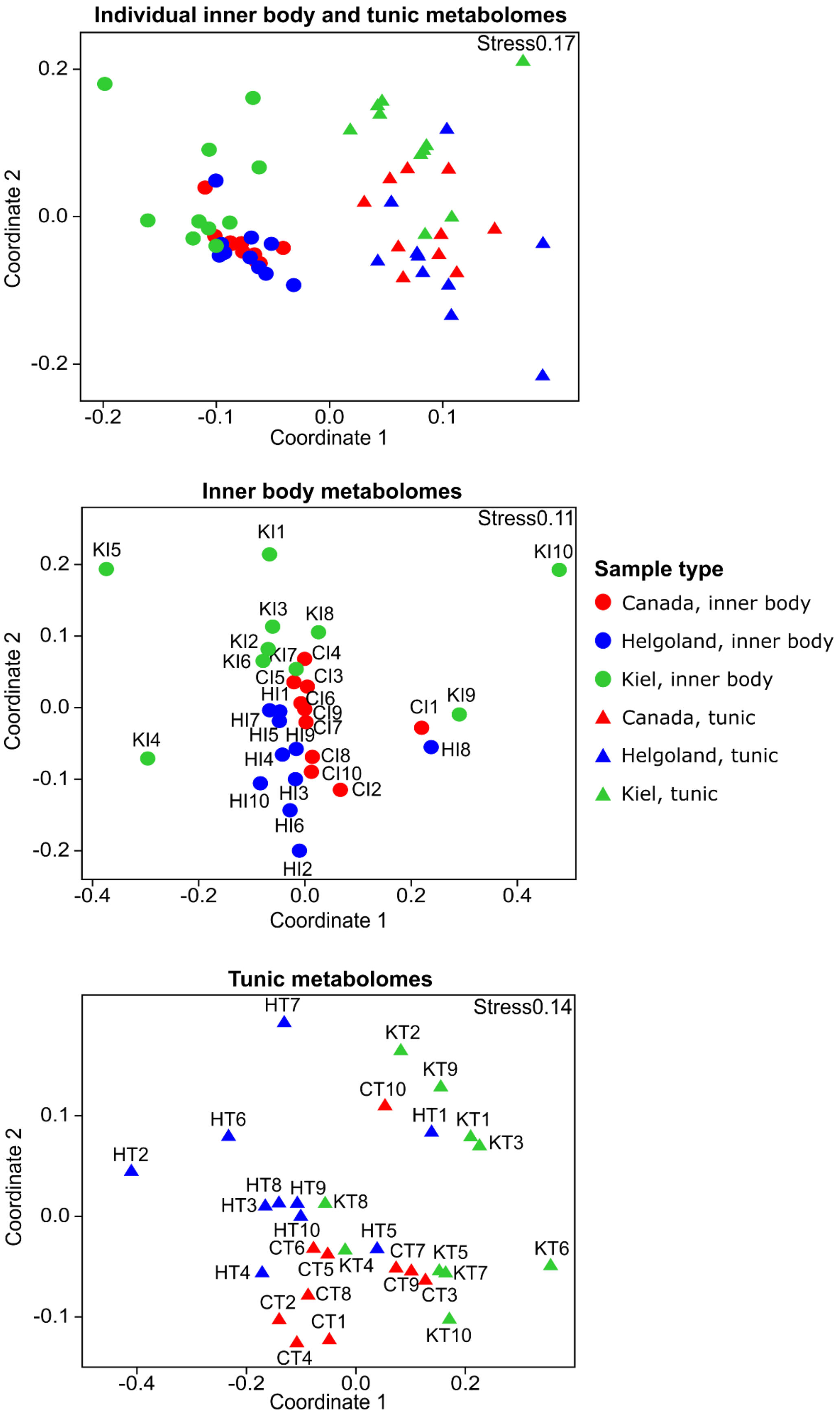

Figure S9. Multivariate ordination plots of UPLC-MS profiles of *C. intestinalis* extracts. NMDS plots are based on a Bray-Curtis similarity matrix. Sampling sites and types are abbreviated as follows: C = Canada, H = Helgoland, K = Kiel; I = inner body, T = tunic.

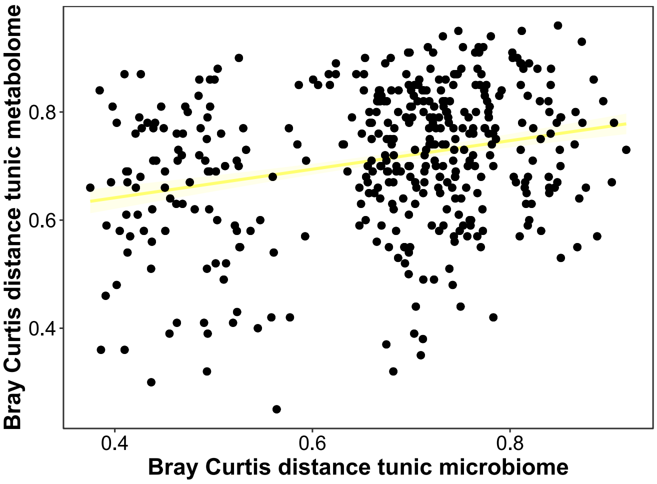

Figure S10. Statistical correlation of individual tunic microbiomes and metabolomes. The regression plot is based on the respective Bray-Curtis similarity matrices of both datasets and the regression line is given with its confidence interval (95%).

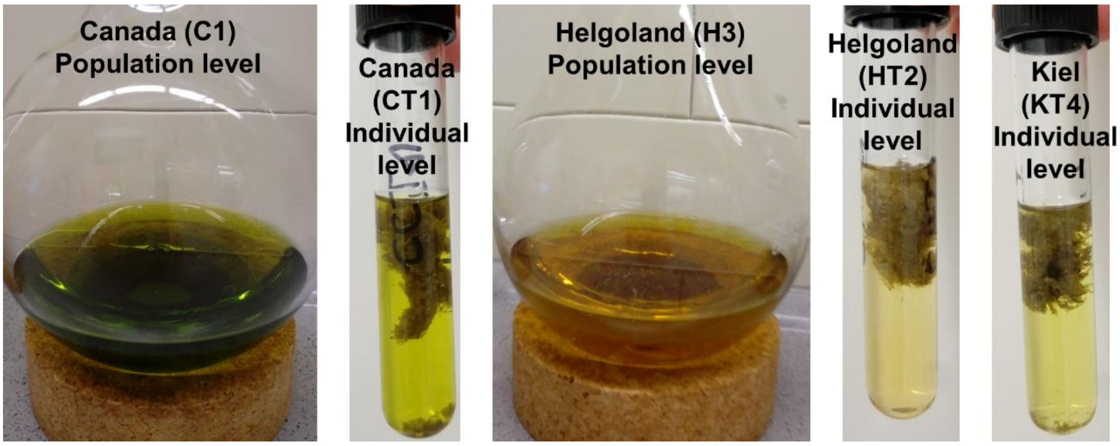

Figure S11. Solvent extracts of different *C. intestinalis* samples. Crude methanol extracts from population and individual level ascidian samples are shown. Sampling sites and types are abbreviated as follows: C = Canada, H = Helgoland, K = Kiel; T = tunic. Pictures by Caroline Utermann.

Table S1. Metadata for microbiome samples analyzed in this study. Sample labels are a combination of the sampling site (C: Canada, H: Helgoland, K: Kiel Fjord) and the respective sample type (G: gut, T: tunic, W: seawater).

Sampling site	Sample type	Replicates (n)	Sample labels
Canada	Ascidian, gut	10	CG1-10
Helgoland		8	HG1-5, HG7, HG9-10
Kiel		9	KG1-9
Canada	Ascidian, tunic	10	CT1-10
Helgoland		10	HT1-10
Kiel		9	KT1-5, KT7-10
Canada	Seawater	3	CW1-3
Helgoland		3	HW1-3
Kiel		3	KW1-3

Table S2. Metadata for metabolome samples analyzed in this study. Sample labels are a combination of the sampling site (C: Canada, H: Helgoland, K: Kiel) and the respective sample type (I: inner body, T: tunic).

Sampling site	Extraction series	Sample type	Replicates (n)	Sample labels
Canada	Population level	Whole animal	3	C1-3
Helgoland			3	H1-3
Kiel			3	K1-3
Canada	Individual level	Ascidian, tunic	10	CT1-10
Helgoland			10	HT1-10
Kiel			10	KT1-10
Canada		Ascidian, inner body	10	CI1-10
Helgoland			10	HI1-10
Kiel			10	KI1-10

Table S3. Parameters of the individual extractions. Samples are given with their dry weight prior extraction and the respective extract weight. Sampling sites and types are abbreviated as follows: C = Canada, H = Helgoland, K = Kiel; I = inner body, T = tunic.

Sample	Dry weight (mg)	Extract (mg)
CI1	33	3.0
CI2	43.6	3.1
CI3	14.8	2.6
CI4	14.5	1.5
CI5	16.2	2.1
CI6	15.3	2.4
CI7	21.4	2.6
CI8	32.8	3.2
CI9	23.3	3.0
CI10	31.1	2.0
CT1	74.2	1.7
CT2	73.1	1.7
CT3	23.7	0.5
CT4	27.9	0.7
CT5	60.5	1.2
CT6	72.4	3.1
CT7	32.8	1.0
CT8	63	2.9
CT9	30.7	0.5
CT10	47.2	1.2
HI1	26.1	5.4
HI2	67.8	12.0
HI3	46.5	14.0
HI4	27.2	5.3
HI5	45.6	6.9
HI6	55.6	11.1
HI7	26.2	9.1
HI8	56.7	8.5
HI9	53	8.4
HI10	46.9	8.5
HT1	14.1	1.4
HT2	137	14.8
HT3	68.4	6.9

Sample	Dry weight (mg)	Extract (mg)
HT4	70.6	4.8
HT5	28.8	0.8
HT6	99.1	8.8
HT7	70.9	8.7
HT8	60.6	4.4
HT9	60.3	4.0
HT10	57.3	4.2
KI1	6.4	0.8
KI2	22.5	2.3
KI3	17.8	0.9
KI4	26.4	2.8
KI5	24.9	1.4
KI6	12.7	0.5
KI7	13.8	1.4
KI8	20.3	1.2
KI9	45.5	2.0
KI10	16.6	1.2
KT1	18.7	0.6
KT2	36.2	1.2
KT3	21.3	0.4
KT4	38.5	1.6
KT5	21.3	0.5
KT6	3.1	0.8
KT7	24.4	0.5
KT8	31.8	1.7
KT9	26.5	1.2
KT10	26.8	0.6

Table S4. Alpha diversity measures of amplicon sequences. The five different indices are given as average values with standard deviation (SD). Sampling sites and types are abbreviated as follows: C = Canada, H = Helgoland, K = Kiel; G = gut, T = tunic, W = seawater.

Group	OTU count (Count)	SD (Count)	Chao1	SD (Chao1)	Faith's Phylogenetic Diversity (PD)	SD (PD)	Shannon (H')	SD (H')	Simpson (D)	SD (D)
CG	337	145	387	181	18.7	6.7	4.5	0.7	0.96	0.03
HG	148	74	181	134	9.7	3.9	3.6	0.4	0.93	0.04
KG	256	89	317	111	14.8	4.6	3.5	1.5	0.79	0.29
CT	506	88	961	183	25.2	3.5	4.1	0.6	0.91	0.07
HT	289	125	535	210	14.1	5.5	3.2	1.0	0.86	0.10
KT	445	168	850	325	22.3	8.0	3.9	1.1	0.88	0.12
CW	308	55	522	127	15.6	2.9	4.1	0.3	0.95	0.02
HW	334	16	601	34	18.4	0.7	4.2	0.1	0.96	0.00
KW	722	52	1381	73	35.7	2.5	5.2	0.3	0.97	0.01
G (all)	247	134	295	169	14.7	6.5	3.9	1.1	0.89	0.19
T (all)	413	159	782	305	20.5	7.6	3.7	1.0	0.88	0.10
W (all)	455	194	835	397	23.3	9.2	4.5	0.5	0.96	0.02
C (G, T)	421	147	674	339	21.9	6.3	4.3	0.7	0.93	0.06
H (G, T)	219	126	358	251	12.1	5.3	3.4	0.8	0.90	0.09
K (G, T)	350	164	583	360	18.6	7.6	3.7	1.4	0.84	0.23

Table S5. Tukey's HSD test comparing observed (OTU count), estimated (Chao1) OTUs, and phylogenetic diversity (PD) detected in ascidian samples at three different sampling sites. Sampling sites and types are abbreviated as follows: C = Canada, H = Helgoland, K = Kiel; G = gut, T = tunic.

Compared groups	p (OTU count)	p (Chao1)	p (PD)
CG x HG	0.045	0.477	0.039
CG x HT	0.993	0.802	0.673
CG x KG	0.869	0.998	0.857
CG x KT	0.607	<0.001	0.901
CT x HG	<0.001	<0.001	<0.001
CT x HT	0.006	0.001	0.002
CT x KG	0.001	<0.001	0.006
CT x KT	0.973	0.959	0.974
HG x KG	0.675	0.911	0.660
HG x KT	<0.001	<0.001	0.001
HT x KG	1.000	0.357	1.000
HT x KT	0.147	0.038	0.061

Table S6. ANOSIM comparison of amplicon sequencing results. ANOSIM was computed based on the Bray-Curtis similarity index in order to statistically compare the different sample groups. Results are given with the respective R and p value.

Test		Compared groups	R	p
Sample type		Gut x Seawater	0.7280	0.0001
		Gut x Tunic	0.7035	0.0001
		Tunic x Seawater	0.9592	0.0001
Sampling site	Gut	Canada x Helgoland	0.9716	0.0001
		Canada x Kiel	0.7331	0.0001
		Helgoland x Kiel	0.9839	0.0001
	Tunic	Canada x Helgoland	0.8362	0.0001
		Canada x Kiel	0.8337	0.0001
		Helgoland x Kiel	0.8038	0.0001

Table S7. Significantly different abundant bacterial phyla. Significance testing was performed with the Kruskal-Wallis-Test. Significant phyla with a relative abundance >0.05% are shown. Sampling sites and types are abbreviated as follows: C = Canada, H = Helgoland, K = Kiel; G = gut, T = tunic, W = seawater.

Phylum	Statistics	p	Relative abundance (%)									
			All	CG	CT	CW	HG	HT	HW	KG	KT	KW
Acidobacteria	24.8	1.7E-03	0.3	0.6	0.4	0.0	0.3	0.1	0.0	0.2	0.5	0.2
Actinobacteria	52.8	1.2E-08	5.4	5.2	0.9	3.5	19.4	0.3	5.1	8.3	1.2	7.7
Bacteroidetes	43.0	8.9E-07	16.6	9.6	21.0	30.8	3.8	19.8	46.3	3.7	21.6	28.3
Chloroflexi	21.5	5.9E-03	1.2	2.7	0.7	0.0	0.5	2.2	0.2	1.1	0.5	0.4
Cyanobacteria	49.0	6.5E-08	8.5	19.0	11.6	1.3	13.9	0.1	1.6	11.0	0.1	8.1
Epsilonbacteraeota	42.9	9.1E-07	3.2	1.5	0.4	0.5	0.7	0.0	0.5	5.8	12.9	3.4
Firmicutes	52.3	1.5E-08	3.9	1.5	0.0	0.1	26.9	0.0	0.3	1.8	0.2	1.0
Patescibacteria	18.4	1.9E-02	0.6	0.8	0.2	0.0	1.0	0.6	0.1	0.6	1.3	0.3
Planctomycetes	29.9	2.2E-04	0.3	0.2	0.3	0.0	0.0	0.4	0.2	0.2	0.4	0.1
Proteobacteria	36.1	1.7E-05	53.8	50.1	60.7	62.4	19.5	74.3	42.0	56.1	58.7	47.6
Spirochaetes	21.8	5.4E-03	0.1	0.1	0.1	0.0	0.0	0.2	0.0	0.1	0.0	0.1
Tenericutes	47.5	1.3E-07	2.3	4.7	0.0	0.0	8.1	0.1	0.0	4.2	0.1	0.3
Verrucomicrobia	25.8	1.2E-03	1.6	1.5	3.2	1.1	0.7	1.6	2.9	0.6	1.8	0.6

Table S8. Significantly different abundant bacterial classes, families and genera. Significance testing was performed with the Kruskal-Wallis-Test. Significant phyla with a relative abundance ≥1% are shown. Sampling sites and types are abbreviated as follows: C = Canada, H = Helgoland, K = Kiel; G = gut, T = tunic, W = seawater.

Taxonomic identification	Statistics	p	Relative abundance (%)									
			**All**	**CG**	**CT**	**CW**	**HG**	**HT**	**HW**	**KG**	**KT**	**KW**
**Class**												
Acidimicrobiia	42.2	1.27E-06	1.6	2.8	0.8	0.8	0.5	0.2	3.9	2.7	1.0	3.5
Actinobacteria	54.6	5.19E-09	3.6	2.2	0.0	2.8	17.5	0.1	1.1	4.9	0.1	3.9
Alphaproteobacteria	48.0	9.95E-08	39.2	35.2	51.7	43.7	15.2	67.6	26.5	18.5	46.4	29.2
Anaerolineae	28.7	3.57E-04	1.1	2.5	0.7	0.0	0.3	2.2	0.0	0.8	0.5	0.3
Bacteroidia	43.2	8.03E-07	16.5	9.5	20.9	30.1	3.8	19.8	46.1	3.7	21.5	28.0
Campylobacteria	40.7	2.38E-06	3.2	1.5	0.4	0.5	0.7	0.0	0.5	5.8	12.9	3.4
Clostridia	52.7	1.25E-08	3.4	1.2	0.0	0.1	23.4	0.0	0.2	1.5	0.2	1.0
Deltaproteobacteria	28.5	3.85E-04	1.9	3.6	1.8	0.2	1.0	1.2	2.2	2.8	1.3	2.4
Gammaproteobacteria	40.3	2.82E-06	12.6	11.2	7.1	18.5	3.2	5.4	13.3	34.7	10.9	16.0
Mollicutes	42.3	1.20E-06	2.3	4.7	0.0	0.0	8.1	0.1	0.0	4.2	0.1	0.3
Oxyphotobacteria	49.3	5.61E-08	8.5	18.9	11.6	1.2	13.8	0.1	1.6	11.0	0.1	8.1
Verrucomicrobiae	26.9	7.41E-04	1.6	1.5	3.2	1.1	0.7	1.6	2.9	0.6	1.8	0.6
**Family**												
Alphaproteobacteria (unclassified)	46.4	2.03E-07	6.0	2.0	12.5	0.0	0.8	17.4	0.4	0.3	6.5	0.1
Arcobacteraceae	37.9	7.88E-06	2.4	0.5	0.4	0.2	0.0	0.0	0.2	3.3	12.6	0.7
Bifidobacteriaceae	50.7	2.96E-08	1.4	0.0	0.0	0.0	11.5	0.0	0.0	0.0	0.0	0.0
Cyanobiaceae	52.9	1.14E-08	5.8	16.7	0.1	1.1	10.7	0.1	1.6	10.5	0.1	7.3
Entomoplasmatales *incertae sedis*	48.0	9.90E-08	2.2	4.5	0.0	0.0	7.9	0.0	0.0	4.1	0.0	0.0
Flavobacteriaceae	43.6	6.78E-07	13.0	7.3	18.2	19.1	2.0	19.2	30.2	2.4	20.0	10.4
Gammaproteobacteria (unclassified)	30.7	1.57E-04	1.0	1.2	2.1	0.1	0.1	0.9	0.4	0.4	1.4	0.5
Halieaceae	36.1	1.70E-05	1.4	1.9	0.9	1.5	0.6	0.3	2.6	2.7	0.7	2.9
Kiloniellaceae	26.1	9.98E-04	1.1	0.1	0.0	0.0	1.8	0.0	0.0	0.0	6.1	0.0
Kordiimonadaceae	53.6	8.11E-09	5.4	0.1	19.5	0.0	0.0	13.0	0.0	0.0	2.6	0.0
Lachnospiraceae	38.4	6.22E-06	1.1	0.1	0.0	0.0	8.6	0.0	0.1	0.1	0.0	0.1
Oxyphotobacteria (unclassified)	55.3	3.94E-09	1.1	0.9	3.0	0.1	3.0	0.0	0.1	0.4	0.0	0.6
Phormidesmiaceae	48.4	8.14E-08	1.1	0.4	7.0	0.0	0.0	0.0	0.0	0.0	0.0	0.1
Pseudomonadaceae	44.9	3.89E-07	2.9	0.0	0.0	0.0	0.0	0.0	0.0	20.6	0.3	0.8
Rhizobiaceae	32.3	8.11E-05	2.9	2.9	2.9	0.1	1.3	4.2	0.0	1.2	6.9	0.3
Rhizobiales (unclassified)	50.0	4.09E-08	1.3	0.0	1.2	0.0	0.0	6.9	0.0	0.1	0.6	0.0
Rhodobacteraceae	41.9	1.42E-06	13.5	24.6	5.4	33.4	8.2	7.3	21.4	9.2	13.7	23.8
S25_593 (Rickettsiales)	47.9	1.03E-07	1.6	0.1	2.2	0.0	0.3	7.4	0.0	0.1	0.7	0.0
Sphingomonadaceae	40.0	3.26E-06	1.4	1.2	1.7	0.0	0.1	3.8	0.0	0.5	2.3	0.3
Terasakiellaceae	26.1	1.02E-03	1.1	0.1	0.9	0.2	0.8	4.0	0.0	0.2	1.1	0.2
**Genus**												
Acrophormium (PCC_7375)	51.1	2.48E-08	1.1	0.3	6.6	0.0	0.0	0.0	0.0	0.0	0.0	0.0
Alphaproteobacteria (unclassified)	46.4	2.03E-07	6.0	2.0	12.5	0.0	0.8	17.4	0.4	0.3	6.5	0.1
Arcobacter	37.9	7.88E-06	2.4	0.5	0.4	0.2	0.0	0.0	0.2	3.3	12.6	0.7
Bifidobacterium	50.7	2.96E-08	1.4	0.0	0.0	0.0	11.5	0.0	0.0	0.0	0.0	0.0

Taxonomic identification	Statistics	p	Relative abundance (%)									
			All	CG	CT	CW	HG	HT	HW	KG	KT	KW
Candidatus Hepatoplasma	48.0	9.90E-08	2.2	4.5	0.0	0.0	7.9	0.0	0.0	4.1	0.0	0.0
Flavobacteriaceae (unclassified)	41.8	1.50E-06	3.2	2.3	6.4	1.2	0.2	6.0	4.4	0.3	3.4	2.3
Gammaproteobacteria (unclassified)	30.7	1.57E-04	1.0	1.2	2.1	0.1	0.1	0.9	0.4	0.4	1.4	0.5
Kiloniella	27.0	7.02E-04	1.1	0.0	0.0	0.0	1.7	0.0	0.0	0.0	6.1	0.0
Kordiimonas	53.6	8.11E-09	5.4	0.1	19.5	0.0	0.0	13.0	0.0	0.0	2.6	0.0
Lentibacter	34.8	2.89E-05	1.3	0.5	0.1	3.6	0.1	0.2	0.1	1.2	4.7	3.8
Oxyphotobacteria (unclassified)	55.3	3.94E-09	1.1	0.9	3.0	0.1	3.0	0.0	0.1	0.4	0.0	0.6
Planktomarina	44.3	5.08E-07	2.1	0.0	0.0	19.0	0.0	0.0	12.7	0.0	0.0	13.2
Pricia	41.6	1.58E-06	4.7	2.0	5.8	0.0	0.0	9.4	0.0	1.0	14.0	0.0
Pseudahrensia	27.2	6.53E-04	1.2	0.6	1.1	0.0	1.2	2.5	0.0	0.3	3.0	0.1
Pseudomonas	44.9	3.89E-07	2.9	0.0	0.0	0.0	0.0	0.0	0.0	20.6	0.3	0.8
Rhizobiales (unclassified)	50.0	4.09E-08	1.3	0.0	1.2	0.0	0.0	6.9	0.0	0.1	0.6	0.0
Rhodobacteraceae (unclassified)	28.2	4.32E-04	4.6	9.1	3.0	1.0	3.2	4.3	1.1	5.3	5.1	2.6
Roseobacter	35.0	2.70E-05	1.6	9.0	0.2	0.1	0.6	0.1	0.0	0.3	0.0	0.1
Rickettsiales group S25_593	47.9	1.03E-07	1.6	0.1	2.2	0.0	0.3	7.4	0.0	0.1	0.7	0.0
Synechococcus (CC9902)	52.7	1.21E-08	5.4	15.8	0.0	1.1	10.6	0.1	1.6	8.9	0.1	6.8

Table S9. Significantly different abundant OTUs. Significance testing across all sample groups was performed with the Kruskal-Wallis-Test for abundant OTUs (≥0.15%). Sampling sites and types are abbreviated as follows: C = Canada, H = Helgoland, K = Kiel; G = gut, T = tunic, W = seawater.

OTU	Phylum	Lowest taxonomic classification	Statistics	p	Relative abundance (%)									
					All	CG	CT	CW	HG	HT	HW	KG	KT	KW
OTU1	Proteobacteria	*Kordiimonas* sp.	53.7	8E-09	5.28	0.13	19.33	0.00	0.00	12.61	0.00	0.01	2.50	0.00
OTU2	Proteobacteria	Alphaproteobacteria	46.5	2E-07	5.60	1.43	11.82	0.00	0.80	16.94	0.00	0.09	6.12	0.02
OTU3	Bacteroidetes	*Pricia* sp.	41.6	2E-06	4.65	2.02	5.65	0.00	0.00	9.27	0.00	1.01	13.78	0.02
OTU4	Proteobacteria	*Pseudomonas* sp.	47.8	1E-07	2.93	0.00	0.00	0.00	0.00	0.00	0.00	20.54	0.31	0.84
OTU5	Epsilonbacteraeota	*Arcobacter* sp.	40.4	3E-06	0.98	0.00	0.00	0.04	0.00	0.00	0.03	0.14	6.89	0.09
OTU6	Cyanobacteria	*Synechococcus* sp. (CC9902)	55.9	3E-09	2.90	9.72	0.03	0.64	8.44	0.05	1.28	1.54	0.03	1.08
OTU7	Proteobacteria	Rickettsiales group S25_593	47.9	1E-07	1.64	0.08	2.25	0.00	0.34	7.39	0.00	0.05	0.69	0.00
OTU8	Cyanobacteria	*Synechococcus* sp. (CC9902)	51.7	2E-08	2.52	6.09	0.02	0.38	2.20	0.01	0.25	7.39	0.05	5.36
OTU9	Proteobacteria	*Kiloniella* sp.	33.3	5E-05	0.83	0.00	0.00	0.00	0.00	0.00	0.00	0.03	5.94	0.00
OTU10	Bacteria (unclassified)	Bacteria (unclassified)	25.9	1E-03	1.48	1.54	0.00	0.00	4.98	0.00	0.00	4.55	0.00	0.00
OTU11	Proteobacteria	*Planktomarina* sp.	44.3	5E-07	1.94	0.03	0.00	17.96	0.00	0.00	11.63	0.00	0.00	12.34
OTU12	Proteobacteria	Terasakiellaceae	43.6	7E-07	0.78	0.03	0.90	0.01	0.04	3.88	0.00	0.00	0.24	0.00
OTU13	Actinobacteria	*Bifidobacterium* sp.	52.3	1E-08	1.13	0.00	0.00	0.00	9.16	0.02	0.01	0.00	0.00	0.00
OTU14	Cyanobacteria	*Acrophormium* sp. (PCC-7375)	53.3	1E-08	0.86	0.27	5.35	0.00	0.00	0.00	0.00	0.00	0.00	0.00
OTU15	Proteobacteria	*Roseobacter* sp.	56.9	2E-09	1.32	8.51	0.02	0.08	0.00	0.01	0.00	0.00	0.00	0.00
OTU16	Tenericutes	*Candidatus* Hepatoplasma	43.0	9E-07	1.22	4.44	0.00	0.00	1.75	0.00	0.00	2.31	0.00	0.00
OTU17	Proteobacteria	*Lentibacter* sp.	35.8	2E-05	1.24	0.46	0.04	3.50	0.07	0.16	0.06	1.17	4.57	3.77
OTU18	Bacteroidetes	Flavobacteriaceae	63.4	1E-10	0.70	0.00	0.00	0.00	0.00	4.55	0.00	0.00	0.00	0.00
OTU19	Proteobacteria	Rhizobiales	46.8	2E-07	0.85	0.00	0.10	0.00	0.00	5.36	0.00	0.00	0.04	0.00
OTU20	Epsilonbacteraeota	*Arcobacter* sp.	53.0	1E-08	0.36	0.00	0.00	0.00	0.00	0.00	0.00	0.64	1.92	0.08

OTU	Phylum	Lowest taxonomic classification	Statistics	p	Relative abundance (%)									
					All	CG	CT	CW	HG	HT	HW	KG	KT	KW
OTU21	Actinobacteria	PeM15	47.2	1E-07	0.70	0.50	0.00	0.05	3.24	0.07	0.93	0.92	0.02	0.88
OTU22	Proteobacteria	*Candidatus* Gigarickettsia	26.1	1E-03	0.51	0.00	0.00	0.00	0.00	0.00	0.00	3.67	0.00	0.00
OTU23	Bacteroidetes	*Formosa* sp.	56.6	2E-09	0.57	0.00	0.00	0.39	0.00	0.00	11.94	0.00	0.00	0.00
OTU24	Epsilonbacteraeota	*Arcobacter* sp.	41.5	2E-06	0.54	0.00	0.00	0.00	0.00	0.00	0.00	1.44	2.44	0.14
OTU25	Tenericutes	*Candidatus* Hepatoplasma	63.6	9E-11	0.75	0.00	0.00	0.00	6.12	0.00	0.00	0.00	0.00	0.00
OTU26	Proteobacteria	Rhizobiales	55.7	3E-09	0.34	0.01	0.98	0.00	0.00	1.26	0.00	0.00	0.00	0.00
OTU27	Proteobacteria	*Pseudahrensia* sp.	41.5	2E-06	0.46	0.16	0.79	0.01	0.00	1.10	0.00	0.06	0.94	0.02
OTU28	Bacteroidetes	*Maritimimonas* sp.	49.4	5E-08	0.44	0.18	2.50	0.00	0.00	0.18	0.00	0.00	0.01	0.00
OTU29	Proteobacteria	*Filomicrobium* sp.	32.9	6E-05	0.39	0.44	0.50	0.00	0.06	0.53	0.01	0.21	0.94	0.03
OTU30	Proteobacteria	*Neptunomonas* sp.	44.9	4E-07	0.40	0.02	0.01	0.00	0.00	0.00	0.00	1.44	1.36	0.07
OTU31	Cyanobacteria	Oxyphotobacteria	56.9	2E-09	0.46	0.50	0.00	0.00	3.04	0.00	0.05	0.00	0.00	0.05
OTU32	Chloroflexi	Ardenticatenaceae	58.9	8E-10	0.28	0.00	0.00	0.00	0.00	1.83	0.00	0.00	0.00	0.00
OTU33	Proteobacteria	*Tateyamaria* sp.	47.3	1E-07	0.42	0.43	0.00	0.03	1.34	0.01	0.12	1.15	0.01	0.40
OTU34	Proteobacteria	*Litoreibacter* sp.	46.2	2E-07	0.35	0.41	0.62	0.00	0.07	1.09	0.00	0.00	0.09	0.00
OTU35	Proteobacteria	Devosiaceae	35.8	2E-05	0.32	0.10	0.71	0.00	0.00	0.66	0.00	0.07	0.58	0.00
OTU36	Firmicutes	*Anaerostipes* sp.	55.0	4E-09	0.37	0.00	0.00	0.00	2.99	0.00	0.03	0.00	0.00	0.00
OTU38	Bacteroidetes	Flavobacteriaceae	44.1	5E-07	0.34	0.15	1.26	0.00	0.00	0.70	0.00	0.01	0.12	0.00
OTU39	Firmicutes	*Romboutsia* sp.	43.5	7E-07	0.42	0.03	0.00	0.00	3.20	0.01	0.00	0.17	0.00	0.01
OTU40	Epsilonbacteraeota	*Sulfurovum* sp.	42.0	1E-06	0.44	0.70	0.01	0.05	0.21	0.00	0.13	1.63	0.08	1.25
OTU41	Proteobacteria	*Sphingorhabdus* sp.	42.9	9E-07	0.30	0.21	0.25	0.00	0.00	1.29	0.00	0.02	0.18	0.00
OTU42	Proteobacteria	*Amylibacter* sp.	40.1	3E-06	0.55	0.01	0.01	7.97	0.01	0.04	2.68	0.00	0.01	0.98
OTU43	Proteobacteria	Rhizobiaceae	28.0	5E-04	0.24	0.18	0.14	0.00	0.00	0.31	0.00	0.06	1.00	0.00
OTU44	Proteobacteria	*Ahrensia* sp.	33.5	5E-05	0.28	0.52	0.55	0.00	0.00	0.36	0.01	0.01	0.42	0.00
OTU45	Bacteroidetes	Flavobacteriaceae	54.0	7E-09	0.29	0.00	0.00	0.00	0.00	0.00	0.00	0.08	2.00	0.03
OTU46	Proteobacteria	*Candidatus* Tenderia	36.6	1E-05	0.25	0.19	0.35	0.02	0.00	0.71	0.00	0.04	0.38	0.03
OTU47	Firmicutes	*Clostridium sensu stricto 1*	49.1	6E-08	0.34	0.00	0.00	0.00	2.77	0.00	0.00	0.00	0.00	0.00
OTU48	Proteobacteria	HOC36	43.1	9E-07	0.34	1.24	0.02	0.01	0.35	0.00	0.00	0.69	0.02	0.09
OTU49	Proteobacteria	*Pseudahrensia* sp.	26.6	8E-04	0.26	0.06	0.09	0.00	0.79	0.79	0.00	0.00	0.15	0.01
OTU50	Firmicutes	*Intestinibacter* sp.	59.5	6E-10	0.30	0.00	0.00	0.00	2.45	0.00	0.00	0.00	0.00	0.00
OTU51	Proteobacteria	*Sphingorhabdus* sp.	40.1	3E-06	0.25	0.05	0.21	0.00	0.00	0.04	0.00	0.19	1.26	0.03
OTU52	Actinobacteria	*Bifidobacterium* sp.	54.1	7E-09	0.27	0.00	0.00	0.00	2.17	0.01	0.00	0.00	0.00	0.00
OTU53	Proteobacteria	Rhodobacteraceae	44.8	4E-07	0.25	0.58	0.13	0.00	0.00	0.01	0.00	0.93	0.08	0.09
OTU54	Proteobacteria	Rhizobiaceae	35.4	2E-05	0.25	0.05	0.04	0.00	0.00	0.00	0.00	0.31	1.36	0.04
OTU55	Proteobacteria	*Ruegeria* sp.	41.7	2E-06	0.28	0.61	0.10	0.00	1.18	0.15	0.00	0.01	0.00	0.00
OTU56	Bacteroidetes	Cryomorphaceae	33.7	5E-05	0.25	0.00	0.00	0.05	0.01	0.00	5.26	0.00	0.00	0.00
OTU57	Proteobacteria	*Pseudahrensia* sp.	22.2	4E-03	0.24	0.02	0.01	0.00	0.00	0.01	0.00	0.10	1.59	0.05
OTU58	Verrucomicrobia	*Rubritalea* sp.	26.1	1E-03	0.20	0.12	1.02	0.00	0.05	0.00	0.02	0.02	0.13	0.00
OTU59	Proteobacteria	Rhodobacteraceae	38.4	6E-06	0.34	2.06	0.07	0.05	0.05	0.01	0.00	0.00	0.00	0.00
OTU61	Proteobacteria	Gammaproteobacteria	48.3	9E-08	0.24	0.27	0.87	0.00	0.00	0.03	0.00	0.01	0.40	0.01
OTU62	Proteobacteria	SAR86 clade	54.7	5E-09	0.33	0.01	0.00	3.93	0.00	0.00	2.99	0.00	0.00	0.22
OTU63	Proteobacteria	Rhodobacteraceae	27.0	7E-04	0.18	0.14	0.23	0.02	0.03	0.67	0.00	0.05	0.06	0.06
OTU64	Proteobacteria	*Ascidiaceihabitans* sp.	58.9	8E-10	0.27	0.00	0.00	0.64	0.00	0.00	4.14	0.00	0.00	1.04
OTU66	Proteobacteria	*Pseudorhodobacter* sp.	39.9	3E-06	0.20	0.19	0.01	0.00	0.00	0.00	0.00	0.19	0.99	0.12
OTU67	Bacteroidetes	Flavobacteriaceae	41.7	2E-06	0.18	0.34	0.80	0.00	0.00	0.02	0.00	0.00	0.00	0.00

OTU	Phylum	Lowest taxonomic classification	Statistics	p	Relative abundance (%)									
					All	CG	CT	CW	HG	HT	HW	KG	KT	KW
OTU68	Firmicutes	*Lachnospiraceae* sp.	58.0	1E-09	0.24	0.00	0.00	0.00	1.97	0.00	0.00	0.00	0.00	0.00
OTU69	Proteobacteria	Rhodobacteraceae	51.5	2E-08	0.20	0.00	0.00	0.00	0.00	0.00	0.00	0.08	1.35	0.05
OTU70	Proteobacteria	PS1 clade	36.9	1E-05	0.15	0.04	0.15	0.00	0.00	0.64	0.00	0.03	0.11	0.00
OTU71	Bacteroidetes	*Psychroserpens* sp.	41.0	2E-06	0.17	0.21	0.37	0.03	0.00	0.32	0.00	0.00	0.17	0.03
OTU72	Proteobacteria	Beijerinckiaceae	37.2	1E-05	0.19	0.11	0.17	0.01	0.00	0.02	0.00	0.05	0.97	0.00
OTU73	Proteobacteria	Rhizobiaceae	32.4	8E-05	0.18	0.17	0.47	0.01	0.01	0.26	0.00	0.07	0.21	0.03
OTU74	Proteobacteria	Rhodobacteraceae	54.0	7E-09	0.31	1.95	0.00	0.07	0.00	0.00	0.00	0.03	0.00	0.00
OTU75	Actinobacteria	*Ilumatobacter* sp.	39.0	5E-06	0.23	0.66	0.07	0.00	0.02	0.01	0.00	0.55	0.06	0.55
OTU76	Bacteroidetes	Flavobacteriaceae	49.2	6E-08	0.19	0.33	0.89	0.01	0.00	0.00	0.00	0.00	0.01	0.00
OTU77	Proteobacteria	*Filomicrobium* sp.	19.0	1E-02	0.17	0.26	0.15	0.00	0.22	0.39	0.00	0.09	0.09	0.00
OTU78	Proteobacteria	Hyphomonadaceae	47.3	1E-07	0.16	0.10	0.75	0.01	0.00	0.18	0.00	0.00	0.02	0.00
OTU79	Proteobacteria	*Kiloniella* sp.	31.2	1E-04	0.21	0.00	0.00	0.00	1.65	0.04	0.00	0.00	0.00	0.00
OTU80	Cyanobacteria	Oxyphotobacteria	43.8	6E-07	0.18	0.01	1.19	0.00	0.00	0.00	0.00	0.00	0.00	0.00
OTU81	Proteobacteria	Rhodobacteraceae	37.7	8E-06	0.15	0.06	0.21	0.00	0.02	0.49	0.00	0.09	0.10	0.00
OTU82	Proteobacteria	Rhizobiaceae	33.3	5E-05	0.16	0.41	0.24	0.02	0.08	0.29	0.00	0.00	0.02	0.00
OTU83	Bacteroidetes	Cryomorphaceae	58.6	9E-10	0.27	0.00	0.00	1.98	0.00	0.00	1.95	0.00	0.00	1.90
OTU85	Proteobacteria	*Anderseniella* sp.	33.9	4E-05	0.18	0.28	0.29	0.00	0.00	0.05	0.00	0.18	0.40	0.11
OTU86	Epsilonbacteraeota	*Arcobacter* sp.	35.4	2E-05	0.19	0.00	0.00	0.00	0.00	0.00	0.00	0.76	0.59	0.04
OTU87	Proteobacteria	Rhizobiaceae	31.3	1E-04	0.15	0.24	0.27	0.00	0.00	0.21	0.00	0.09	0.19	0.04
OTU88	Actinobacteria	*Ilumatobacter* sp.	40.3	3E-06	0.16	0.36	0.05	0.00	0.11	0.01	0.49	0.37	0.02	0.18
OTU89	Bacteroidetes	*Formosa* sp.	64.0	8E-11	0.19	0.00	0.00	0.41	0.00	0.00	3.73	0.00	0.00	0.00
OTU90	Bacteroidetes	NS3a marine group	59.0	7E-10	0.33	0.00	0.00	4.91	0.00	0.00	0.16	0.00	0.00	2.09
OTU91	Bacteroidetes	*Ulvibacter* sp.	44.3	5E-07	0.15	0.18	0.27	0.00	0.00	0.00	0.00	0.11	0.43	0.03
OTU92	Proteobacteria	SUP05 cluster	46.0	2E-07	0.19	0.00	0.00	0.00	0.00	0.00	0.00	1.10	0.23	0.01
OTU93	Proteobacteria	*Altererythrobacter* sp.	32.7	7E-05	0.15	0.03	0.07	0.00	0.00	0.63	0.00	0.01	0.22	0.05
OTU94	Proteobacteria	Sphingomonadaceae	35.8	2E-05	0.15	0.06	0.17	0.00	0.00	0.30	0.00	0.20	0.26	0.00
OTU95	Proteobacteria	Rhodobacteraceae	24.8	2E-03	0.15	0.18	0.06	0.00	0.12	0.00	0.00	0.62	0.10	0.05
OTU96	Bacteroidetes	*Tenacibaculum* sp.	31.4	1E-04	0.16	0.17	0.17	0.00	0.77	0.06	0.02	0.00	0.00	0.00
OTU98	Actinobacteria	*Mycobacterium* sp.	49.5	5E-08	0.18	0.27	0.01	0.02	0.00	0.00	0.00	0.75	0.02	0.58
OTU99	Proteobacteria	Rhodobacteraceae	34.6	3E-05	0.15	0.14	0.25	0.01	0.00	0.23	0.00	0.02	0.33	0.07
OTU100	Bacteroidetes	Bacteroidia	37.2	1E-05	0.15	0.00	0.00	0.00	1.14	0.07	0.00	0.00	0.00	0.00
OTU103	Proteobacteria	*Candidatus* Puniceispirillum	64.0	8E-11	0.24	0.00	0.00	2.99	0.00	0.00	0.84	0.00	0.00	1.40
OTU104	Firmicutes	*Terrisporobacter* sp.	63.6	9E-11	0.15	0.00	0.00	0.00	1.17	0.00	0.01	0.00	0.00	0.00
OTU105	Proteobacteria	*Halioglobus* sp.	33.3	5E-05	0.15	0.11	0.01	0.00	0.12	0.01	0.00	0.71	0.03	0.23
OTU106	Proteobacteria	*Ruegeria* sp.	36.9	1E-05	0.16	0.76	0.11	0.00	0.16	0.04	0.00	0.00	0.00	0.00
OTU114	Firmicutes	*Dorea* sp.	37.4	1E-05	0.17	0.00	0.00	0.00	1.36	0.01	0.01	0.00	0.00	0.00
OTU123	Proteobacteria	Rhodobacteraceae	30.9	1E-04	0.15	0.34	0.01	0.00	0.31	0.00	0.00	0.37	0.02	0.05
OTU161	Bacteroidetes	NS5 marine group	63.9	8E-11	0.17	0.00	0.01	2.71	0.00	0.00	0.49	0.00	0.00	0.45

Table S10. Classification of abundant OTUs detected in this study. Abundant OTUs: ≥1% of overall relative abundance. Rel. abund. = Relative abundance, Id. = Identity, Ubc = uncultured bacterium clone, Uncult. = Uncultured.

OTU	Rel. abund. (%)	Silva classification (lowest taxonomic rank; class, genus)	Next related hit according to BLAST				Lowest taxonomic classification according to BLAST			
			Classification	Accession no.	Id. (%)	Source	Classification	Accession no.	Id. (%)	Source
OTU1	5.3	Alphaproteobacteria, *Kordiimonas*	Ubc SanDiego_a6487	KF799727.1	98.76	Ascidian (*Ciona intestinalis*; gut)	*Kordiimonas* sp.	KF494349.1	98.76	Ascidian (*Ciona intestinalis*; tunic)
OTU2	5.6	Alphaproteobacteria, unclassified	Ubc Woods-Hole_a4133	KF799375.1	98.75	Ascidian (*Ciona intestinalis*; gut)	Rhizobiales	e.g. MN006421.1	93.27	Various
OTU3	4.7	Bacteroidia, *Pricia*	Ubc Woods-Hole_a5311	KF799010.1	98.57	Ascidian (*Ciona intestinalis*; gut)	*Arenibacter* sp.	KF494352.1	98.57	Ascidian (*Ciona intestinalis*; tunic)
OTU4	2.9	Gammaproteobacteria, *Pseudomonas*	*Pseudomonas* sp.	MH244157.1	99.30	Sediment				
OTU6	2.9	Oxyphotobacteria, *Synechococcus*_CC9902	Ubc DNA47	MG011059.1	99.75	Krill (*Euphausia mucronata*; stomach)	*Synechococcus* sp.	MH358353.1	99.75	Marine environment
OTU7	1.6	Alphaproteobacteria, unclassified (S25-593 group)	Ubc SanDiego_a6337	KF799711.1	99.01	Ascidian (*Ciona intestinalis*; gut)	Uncult. alphaproteo-bacterium_1-21	FJ659126.1	95.04	Ascidian (*Aplidium conicum*; tunic)
OTU8	2.5	Oxyphotobacteria, *Synechococcus*_CC9902	Ubc HAMb1_059	JX983984.1	98.77	Marine biofilm	*Synechococcus* sp.	KU867940.1	98.52	Seawater
OTU10	1.5	Unclassified (Bacteria)	Ubc Woods-Hole_a5143	KF798938.1	99.51	Ascidian (*Ciona intestinalis*; gut)	n.a. (only 76% identity)			
OTU11	1.9	Alphaproteobacteria, *Planktomarina*	Uncult. alphaproteobacterium clone PI_4d12b	AY580449.1	99.26	Seawater	Rhodo-bacteraceae	KU173743.1 or NR_125550.1	99.01	Seawater
OTU13	1.1	Actinobacteria, *Bifidobacterium*	*Bifidobacterium dentium*	LR134349.1	100	Human (Dental Caries)				
OTU15	1.3	Alphaproteobacteria, *Roseobacter*	Marine bacterium BPY-W9	AB562975.1	98.01	Red algae (*Porphyra yezoensis*, Japan)	*Roseobacter* sp.	MK224709.1	97.27	Red algae (*Neogoniolithon brassica-florida*)
OTU16	1.2	Mollicutes, *Candidatus_Hepatoplasma*	Ubc Woods-Hole_a5449	KF799049.1	91.08	Ascidian (*Ciona intestinalis*; gut)	n.a. (only 82% identity)			
OTU17	1.2	Alphaproteobacteria, *Lentibacter*	Uncult. marine bacterium, clone 85PALMAR09	HE981604.1	99.50	Seawater	*Litoreibacter* sp.	KJ513684.1	99.26	Seawater

Table S11. Putative annotation of metabolites detected in *C. intestinalis* bulk extracts (population level). Each detected compound is given with the experimentally determined *m/z* value. Putative molecular formulae were calculated by the elemental composition tool in the MassLynx software. The dereplication tool(s) (Derep. tool(s)) used to annotate the compounds are DNP (Dictionary of Natural Products [2]), GNPS (Dereplication workflow available at Global Natural Product Social Molecular Networking [1]), ISDB-UNPD (*in silico* MS/MS database of the Universal Natural Product Database [3]), ML (MarinLit [4]) and MN (molecular networking [1]). Occurrence of abundant peaks (detected in ≥5 replicates) in individual extracts is given with the respective number of replicates for inner body (IB) and tunic (T) extracts separately. R_t: Retention time. IC: Identification confidence level after Sumner et al. 2007 [5]. Nf: No fragmentation pattern detected. ↑: Metabolite production was specifically enhanced (at least 10-fold larger peak area) in the respective sampling location (sampling locations are abbreviated: C = Canada, H = Helgoland, K = Kiel). Underlined occurrence: enhancement/specificity was manually detected from MS chromatograms (peak intensity), since compound was not in automatic peak list. Refs: references.

Peak no.	*m/z* $[M+H]^+$	R_t (min)	Putative molecular formula	IC	Fragmentation pattern	Putative identification	Derep. tool(s)	Chemical family	Biological origin	Occurrence	IB	T	Refs
1	248.161	3.04	$C_{10}H_{21}N_3O_4$	4	124.0692					H3, K1	5	6	
2	466.2826	3.10	$C_{26}H_{35}N_5O_3$	4	Nf					K↑			
3	386.3221	3.10	$C_{13}H_{39}N_9O_4$	4	Nf					K↑			
4	361.3556	3.10	$C_{19}H_{44}N_4O_2$	4	140.1747, 157.2030, 290.3256					not C	13	9	
5	429.4159	3.10	$C_{29}H_{52}N_2$	2	72.1032, 140.1747, 155.1360, 220.0516, 228.2256, 340.2377, 351.1815	Halichonine B	ISDB-UNPD	Sesquiterpene alkaloid	Sponge: *Halichondria okadai*	not C	13	7	[6]
6	517.2661	3.12	$C_{31}H_{48}O_6$	3	345.2962, 363.3029, 439.3996		DNP, ML	Sterol	Various marine invertebrates, e.g. sponge (*Dysidea herbacea*), coral (*Nephthea bayeri*)	H3, K1			[7], [8]
7	211.0554	3.28	$C_5H_{10}N_2O_7$	4	193.0851					H3, K1	5	8	
8	667.4767	3.31	$C_{35}H_{58}N_{10}O_3$	4	Nf								
9	335.1791	3.45	$C_{26}H_{22}$	4	Nf						7	1	
10	480.3527	3.46	$C_{33}H_{41}N_3$	4	318.3422					C↑	21	18	
11	480.3527	3.52	$C_{33}H_{41}N_3$	4	318.3422						4	6	
12	278.2424	3.69	$C_{18}H_{31}NO$	2	250.2857, 262.3000	Crucigasterin 277	ISDB-UNPD	Polyunsaturated amino alcohol	Ascidian: *Pseudodistoma crucigaster*	H↑	16	2	[9]
13	545.296	3.77	$C_{27}H_{44}O_{11}$	4	373.3291, 391.3394					H3, K1	3	7	
14	280.2590	3.74	$C_{18}H_{33}NO$	2	Nf	Crucigasterin E	ISDB-UNPD	Polyunsaturated amino alcohol	Ascidian: *Pseudodistoma crucigaster*		18	3	[10]
15	310.2712	3.92	$C_{19}H_{35}NO_2$	2	81.0943, 86.0830, 95.1129, 135.1490, 145.1371, 257.2605, 274.2864	D-erythro-4,8,10-sphingatrienine	ISDB-UNPD	Glycosphingolipid	Sea cucumber: *Stichopus variegates*	H only			[11]
16	413.3359	3.97	$C_{19}H_{40}N_8O_2$	4	Nf					H3, K1	1	6	
17	554.5435	4.20	$C_{37}H_{67}N_3$	4	72.1018, 137.1615, 197.2366, 276.3089, 279.3187, 333.3840					H only	7	3	

Peak no.	*m/z* [M+H]$^+$	R_t (min)	Putative molecular formula	IC	Fragmentation pattern	Putative identification	Derep. tool(s)	Chemical family	Biological origin	Occurrence	IB	T	Refs
18	340.2194	4.42	$C_{12}H_{29}N_5O_6$	4	Nf					C↑			
19	468.3076	4.41	$C_{24}H_{41}N_3O_6$	4	Nf						26	4	
20	349.1927	4.57	$C_{10}H_{28}N_4O_9$	4	Nf						18	13	
21	454.3291	4.73	$C_{25}H_{39}N_7O$	4	104.1345					not K	24	3	
22	317.1698	4.81	$C_{10}H_{20}N_8O_4$	4	Nf								
23	349.1927	4.84	$C_{10}H_{28}N_4O_9$	4	Nf					H3, K1	3	4	
24	277.2130	4.86	$C_{17}H_{24}O_3$	2	107.1138, 121.1301, 135.1430, 149.1648, 195.9527	Spirodysin	ISDB-UNPD	Sesquiterpenoid	Sponge: *Dysidea* sp.		17	2	[12]
25	599.4107	4.92	$C_{37}H_{58}O_4S$	3	221.1886, 507.3958		MN	Alkyl sulfate		H3, K1	0	8	
26	301.2123	4.98	$C_{15}H_{28}N_2O_4$	2	187.1823, 265.2364, 283.2443	Lipoamide A	DNP, ML	Lipoamide	Bacterium: *Bacillus pumilus*		27	21	[13]
27	568.3410	5.20	$C_{20}H_{49}N_5O_{13}$	4	Nf						24	13	
28	520.3409	5.20	$C_{33}H_{45}NO_4$	4	Nf					C only	10	11	
29	488.2342	5.20	$C_{20}H_{33}N_5O_9$	4	Nf						22	23	
30	453.2224	5.20	$C_{18}H_{28}N_8O_6$	4	Nf					H3	21	23	
31	468.3430	5.27	$C_{25}H_{45}N_3O_5$	4	104.1349, 427.2712, 363.9669						29	21	
32	842.5110	5.35	$C_{38}H_{63}N_{15}O_7$	4	Nf					C only	3	8	
33	480.3438	5.36	$C_{26}H_{45}N_3O_5$	4	Nf					not H	18	26	
34	454.2899	5.47	$C_{19}H_{35}N_9O_4$	4	313.3146					not K	18	0	
35	376.2775	5.47	$C_{14}H_{33}N_9O_3$	3	Nf		MN	Tetrapyrrole		C only			
36	506.4057	5.54	$C_{27}H_{55}NO_7$	4	104.1349					H↑	11	8	
37	235.1639	5.57	$C_6H_{18}N_8O_2$	4	Nf					C↑			
38	542.3815	5.57	$C_{29}H_{47}N_7O_3$	4	Nf					C only	9	0	
39	512.3700	5.61	$C_{27}H_{49}N_3O_6$	4	104.135						10	19	
40	321.2397	5.67	$C_{15}H_{32}N_2O_5$	4	151.1442					H↑	2	7	
41	270.3127	5.69	$C_{18}H_{39}N$	3	Nf		MN	Tetrapyrrole		H only			
42	496.3393	5.72	$C_{27}H_{41}N_7O_2$	4	104.1346, 184.1077, 478.3760						27	21	
43	438.2986	5.88	$C_{27}H_{39}N_3O_2$	2	266.3131, 284.3345, 420.3313	Lyngbyatoxin A	ISDB-UNPD	Indole alkaloid	E.g. Cyanobacterium: *Moorea producens*		15	8	[14]
44	276.2263	5.94	$C_9H_{25}N_9O$	4	Nf					C↑	4	2	
45	494.3591	5.94	$C_{27}H_{47}N_3O_5$	4	104.1345, 184.1088						23	18	
46	522.3537	6.08	$C_{28}H_{47}N_3O_6$	4	104.1343, 184.1078, 504.3933						27	22	
47	480.3438	6.14	$C_{21}H_{46}N_7O_3Cl$	4	Nf					not H	20	27	
48	985.7030	6.18	$C_{64}H_{92}N_2O_6$	4	Nf					H only			
49	452.3128	6.46	$C_{28}H_{41}N_3O_2$	2	280.3400, 298.3514, 434.3490	Blastmycetin E	ISDB-UNPD	Indole alkaloid	Bacterium: *Streptoverticillium blastmyceticum*		28	22	[15]
50	510.3580	6.49	$C_{26}H_{56}NO_6P$	2	104.1346, 184.1077, 327.3210	Lyso-platelet-activating factor (C18)	GNPS	Phospholipid	Sponge: *Spirastrella purpurea*		19	6	[16]
51	506.3597	6.50	$C_{23}H_{48}N_7O_3Cl$	4	Nf					not H	13	17	

Peak no.	*m/z* $[M+H]^+$	R_t (min)	Putative molecular formula	IC	Fragmentation pattern	Putative identification	Derep. tool(s)	Chemical family	Biological origin	Occurrence	IB	T	Refs
52	508.3776	6.55	$C_{29}H_{45}N_7O$	4	104.1350, 184.1079					H↑	13	11	
53	452.3128	6.63	$C_{28}H_{41}N_3O_2$	2	280.3400, 298.3514, 434.3490	Blastmycetin E	ISDB-UNPD	Indole alkaloid	Bacterium: *Streptoverticillium blastmyceticum*		27	14	[15]
54	302.2427	6.70	$C_{11}H_{27}N_9O$	4	Nf					C only	5	2	
55	588.3503	6.70	$C_{29}H_{45}N_7O_6$	2	Nf	MIP-A3	DMNP	Linear peptide	Snail: *Achatina fulica*	C only	9	7	[17]
56	581.4004	6.75	$C_{40}H_{52}O_3$	2	221.1906, 489.3869	α-Doradecin	DNP	Carotenoid	Crab: *Chiromantes haematocheir*	H3, K1	0	6	[18]
57	494.3591	6.77	$C_{27}H_{47}N_3O_5$	4	Nf						20	23	
58	452.3128	6.77	$C_{28}H_{41}N_3O_2$	4	Nf	Blastmycetin E	ISDB-UNPD	Indole alkaloid	Bacterium: *Streptoverticillium blastmyceticum*				[15]
59	428.3738	6.77	$C_{25}H_{49}NO_4$	2	Nf	(4E)-N-[(2R)-1-hydroxy-3-methoxypropan-2-yl]-7-methoxyicos-4-enamide	DNP, ML	Lipopeptide	Cyanobacterium (not identified)		12	6	[19]
60	619.3134	6.77	$C_{33}H_{46}O_{11}$	2	Nf	Antibiotic YM 47525	DNP	Sesquiterpenoid	Fungus (not identified)	C only			[20]
61	344.3479	6.91	$C_{17}H_{41}N_7$	4	Nf						28	27	
62	508.3776	6.91	$C_{29}H_{45}N_7O$	4	104.1351					not H	18	25	
63	482.3227	6.91	$C_{25}H_{43}N_3O_6$	4	Nf						26	3	
64	254.2425	6.99	$C_{16}H_{31}NO$	3	184.2121, 219.2468, 237.2642	a: Crucigasterin D, b: Obscuraminol C	DNP	Polyunsaturated amino alcohol	Ascidian: *Pseudodistoma crucigaster* (a) or *Pseudodistoma obscurum* (b)		2	6	(a) [10], (b) [21]
65	377.2661	7.03	$C_{19}H_{32}N_6O_2$	4	201.2046, 285.2580					H↑	13	1	
66	349.2691	7.03	$C_{17}H_{36}N_2O_5$	4	Nf					H↑	3	10	
67	494.3591	7.03	$C_{27}H_{47}N_3O_5$	4	Nf					C↑	16	13	
68	639.4067	7.12	$C_{30}H_{58}N_2O_{12}$	4	Nf					C only	1	13	
69	599.4107	7.12	$C_{40}H_{54}O_4$	2	109.1296, 185.1529, 233.1781, 341.3246, 544.3911	a: Crassostreaxanthin A, b: Crassostreaxanthin B	DNP, ML	Carotenoid	Bivalve: *Crassostrea gigas*	C↑			[22]
70	466.3312	7.12	$C_{30}H_{43}NO_3$	4	448.3555, 312.3705, 294.3555								
71	370.3654	7.12	$C_{19}H_{43}N_7$	4	Nf						14	10	
72	277.2130	7.21	$C_{13}H_{28}N_2O_4$	4	Nf						26	19	
73	524.3705	7.32	$C_{26}H_{54}NO_7P$	2	104.1354, 184.1063, 341.3518, 506.4213	Platelet-activating factor (PAF)	GNPS	Phospholipid	Various types of cells and animals		27	17	[23]
74	466.3268	7.41	$C_{25}H_{43}N_3O_5$	4	294.3566, 312.3678, 448.3652						29	23	
75	627.3563	7.57	$C_{36}H_{50}O_9$	2	469.3168, 367.3693, 283.0604	Milbemycin α20	DNP	Macrolide	Bacterium: *Streptomyces hygroscopicus* subsp. *aureolacrimosus*				[24]
76	550.3897	7.63	$C_{35}H_{51}NO_4$	4	104.1348, 184.1081, 532.4282						27	21	
77	597.2744	7.63	$C_{23}H_{36}N_{10}O_9$	4	Nf						4	2	

Peak no.	*m/z* [M+H]+	R_t (min)	Putative molecular formula	IC	Fragmentation pattern	Putative identification	Derep. tool(s)	Chemical family	Biological origin	Occurrence	IB	T	Refs
78	376.3172	7.63	$C_{19}H_{41}N_3O_4$	4	Nf					C↑	1	12	
79	403.2805	7.76	$C_{19}H_{39}NaO_5S$	2	293.2614, 311.2804	Sodium 10-(hydroxymethyl)-2,6,14-trimethylpentadecyl sulfate	ML	Alkyl sulfate	Ascidian: *Ciona edwardsii*	H↑	13	2	[25]
80	552.4021	7.76	$C_{31}H_{49}N_7O_2$	4	Nf					C only	1	6	
81	441.2969	7.76	$C_{23}H_{40}N_2O_6$	4	Nf					H↑	8	5	
82	508.3776	7.83	$C_{29}H_{45}N_7O$	4	104.1351					not H	12	27	
83	303.2285	7.91	$C_{15}H_{30}N_2O_4$	4	Nf						29	30	
84	320.2177	8.14	$C_{14}H_{29}N_3O_5$	3	140.1022, 166.0831, 302.2524					H3, K1	4	7	
85	451.2999	8.51	$C_{33}H_{38}O$	4	Nf					H3	8	1	
86	317.2417	8.51	$C_{12}H_{28}N_8O_2$	4	235.3066						15	11	
87	305.2441	8.55	$C_{19}H_{32}N_2O$	2	163.1834	Ikimine A	ISDB-UNPD	Alkylpyridine	Sponge (not identified)		4	6	[26]
88	329.2441	8.60	$C_{17}H_{32}N_2O_4$	2	175.1824, 215.2166, 311.2789	Lipoamide C	DNP, ML	Lipoamide	Bacterium: *Bacillus pumilus*		17	22	[13]
89	305.2441	8.79	$C_{19}H_{32}N_2O$	2	163.1834	Ikimine A	ISDB-UNPD	Alkylpyridine	Sponge (not identified)				[26]
90	609.2709	8.87	$C_{35}H_{36}N_4O_6$	2	531.2986, 559.2847, 591.3218	10-hydroxyphaeophorbide a	DNP	Tetrapyrroles	E.g. ascidian: *Trididemnum solidum*	C↑	7	15	[27]
91	289.2116	9.00	$C_{14}H_{28}N_2O_4$	4	215.2158, 229.2335, 239.2176, 257.2284					H only			
92	625.2678	9.02	$C_{36}H_{33}N_8O_3$	4	538.3119, 566.3060, 581.3383, 608.3168					C only	2	15	
93	400.4145	9.09	$C_{25}H_{53}NO_2$	4	Nf						27	30	
94	603.2276	9.09	$C_{27}H_{43}N_2O_8Br$	4	501.2975, 527.2592, 529.2766					not K			
95	331.2587	9.10	$C_{22}H_{34}O_2$	2	Nf	Clupanodonic acid	ISDB-UNPD	Unsaturated fatty acid	Fish oil	not K	12	13	[28]
96	609.2709	9.28	$C_{35}H_{36}N_4O_6$	2	531.2986, 559.2847, 591.3218	10-hydroxyphaeophorbide a	DNP	Tetrapyrrole	E.g. ascidian: *Trididemnum solidum*	C↑, not K	10	16	[27]
97	641.4202	9.36	$C_{26}H_{56}N_8O_{10}$	4	Nf					C only	1	12	
98	681.4148	9.36	$C_{28}H_{56}N_8O_{11}$	4	Nf					C only	0	10	
99	581.4004	9.36	$C_{40}H_{52}O_3$	2	109.1293, 147.1132, 149.1319, 157.1320, 185.1702, 197.1720, 237.1925, 355.2922	Trikentriorhodin	DNP	Carotenoid	Sponge: *Trikentrion helium*	<u>C only</u>	0	11	[29]
100	658.4239	9.37	$C_{33}H_{39}NO_{13}$	2	109.1300, 127.130, 223.1872	Rubomycin M	DNP	Anthracycline glycoside	Bacterium: *Streptomyces coeruleorubidus*	C only	1	12	[30]
101	291.2291	9.60	$C_{19}H_{30}O_2$	3	121.1306,135.1477, 149.1646, 163.1814, 241.2334, 259.2451		DNP, ML	Unsaturated fatty acid	Various marine origins, e.g. sponge (e.g. *Stelletta* sp.) and alga	H3, K1	4	11	[31], [32]

Peak no.	*m/z* $[M+H]^+$	R_t (min)	Putative molecular formula	IC	Fragmentation pattern	Putative identification	Derep. tool(s)	Chemical family	Biological origin	Occurrence	IB	T	Refs
									(e.g. *Lobophora variegata*)				
102	348.2455	9.68	$C_{23}H_{29}N_3$	2	140.1022, 166.0836, 330.2850						5	13	
103	593.2771	9.69	$C_{35}H_{36}N_4O_5$	2	533.3050	Pheophorbide A	DNP	Tetrapyrrole	E.g. ascidian: *Trididemnum solidum*	C↑	26	11	[27]
104	317.2454	10.18	$C_{21}H_{32}O_2$	2	267.2503, 285.2617	a: 5E,7E,9E,14Z,17Z-eicosapentaenoic acid, b: 5Z,7E,9E,14Z,17Z-eicosapentaenoic acid	ISDB-UNPD	Unsaturated fatty acid	Alga: *Ptilota filicina*		2	15	[33]
105	535.2693	10.25	$C_{33}H_{34}N_4O_3$	2	507.3204	Pyropheophorbide A	DNP, ML	Tetrapyrrole	E.g. bivalve: *Ruditapes philippinarum*		28	29	[34]
106	293.2431	10.44	$C_{14}H_{32}N_2O_4$	4	81.0950, 95.1122, 109.1295, 123.1465, 137.1631, 243.2490, 261.2608						2	3	
107	363.2984	10.46	$C_{24}H_{42}O_2$	2	Nf	Strongylodiol G	ISDB-UNPD	Acetylenic alcohol	Sponge: *Petrosia* sp.		22	11	[35]
108	565.2440	10.48	$C_{33}H_{32}N_4O_5$	2	Nf	Purpurin 18	DNP	Tetrapyrrole	E.g. bivalve: *Ruditapes philippinarum*		18	0	[34]
109	681.4148	10.48	$C_{44}H_{56}O_6$	4	Nf					C only	4	12	
110	639.3239	10.64	$C_{43}H_{38}N_6$	4	566.3386. 579.3464					H3, K1	1	6	
111	343.2594	10.71	$C_{23}H_{34}O_2$	2	269.2657, 293.2670, 311.2781	Docosahexaenoic acid methyl ester	ISDB-UNPD	Unsaturated fatty acid	Ascidian: *Pseudodistoma aureum*		7	13	[36]
112	647.5734	10.89	$C_{43}H_{76}O_2$	4	Nf					H only			
113	732.5054	10.99	$C_{42}H_{63}N_9O$	4	Nf						11	25	
114	518.4949	11.30	$C_{34}H_{63}NO_2$	4	250.2922, 262.2921, 280.3038					H↑	15	2	
115	793.4943	11.60	$C_{53}H_{64}N_2O_4$	4	Nf					C only	1	9	
116	564.3982	11.79	$C_{27}H_{49}N_9O_4$	4	Nf					C only	0	14	
117	549.2866	11.91	$C_{34}H_{36}N_4O_3$	2	461.2792, 436.2999	Methyl pyropheophorbide a	DNP	Tetrapyrrole	E.g. cyanobacterium: *Spirulina maxima*	H3, K1	1	13	[37]
118	748.5408	11.99	$C_{35}H_{75}N_5O_{10}$	4	Nf						28	10	
119	573.1493	11.99	$C_{24}H_{29}N_2O_{12}Cl$	4	135.1505, 554.5661					C↑			
120	546.4907	11.99	$C_{35}H_{63}NO_3$	2	Nf	Bacteriohopane-aminotriol	DNP	Hopanoid	Bacterium: e.g. *Rhodopseudomonas acidophila*		19	1	[38]
121	543.1011	12.10	$C_{21}H_{21}N_6O_8Cl$	4	Nf					C only			

Table S12. ANOSIM comparison of UPLC-MS/MS profiles of *C. intestinalis* extracts. ANOSIM calculations were based on the Bray-Curtis similarity index and results are given with the respective R score and p value.

Test	Extraction procedure	Tissue(s)	Compared groups	R	p
Sampling sites (all metabolites)	Population level	Whole body	Canada x Helgoland	0.6667	0.1
			Canada x Kiel	0.5556	0.09
			Helgoland x Kiel	0.8889	0.1
Sampling sites (core metabolites only)			Canada x Helgoland	1	0.1
			Canada x Kiel	1	0.1
			Helgoland x Kiel	1	0.1
Tissue	Individual level	Inner body & tunic	Inner body x tunic	0.6768	0.0001
Sampling sites		Inner body	Canada x Helgoland	0.2502	0.0025
			Canada x Kiel	0.2342	0.0011
			Helgoland x Kiel	0.3184	0.0003
		Tunic	Canada x Helgoland	0.2484	0.0049
			Canada x Kiel	0.3562	0.0027
			Helgoland x Kiel	0.4518	0.0012

Supplementary References

1. Wang, M.; Carver, J.J.; Phelan, V.V.; Sanchez, L.M.; Garg, N.; Peng, Y.; Nguyen, D.D.; Watrous, J.; Kapono, C.A.; Luzzatto-Knaan, T.; et al. Sharing and community curation of mass spectrometry data with Global Natural Products Social Molecular Networking. *Nat. Biotechnol.* **2016**, *34*, 828-837, doi:10.1038/nbt.3597.
2. Blunt, J.W.; Munro, M.H. *Dictionary of marine natural products with CD-ROM*. CRC Press: 2007.
3. Allard, P.M.; Peresse, T.; Bisson, J.; Gindro, K.; Marcourt, L.; Pham, V.C.; Roussi, F.; Litaudon, M.; Wolfender, J.L. Integration of molecular networking and *in-silico* MS/MS fragmentation for natural products dereplication. *Anal. Chem.* **2016**, *88*, 3317-3323, doi:10.1021/acs.analchem.5b04804.
4. Blunt, J.W.; Copp, B.R.; Keyzers, R.A.; Munro, M.H.G.; Prinsep, M.R. Marine natural products. *Nat. Prod. Rep.* **2017**, *34*, 235-294, doi:10.1039/c6np00124f.
5. Sumner, L.W.; Amberg, A.; Barrett, D.; Beale, M.H.; Beger, R.; Daykin, C.A.; Fan, T.W.; Fiehn, O.; Goodacre, R.; Griffin, J.L.; et al. Proposed minimum reporting standards for chemical analysis Chemical Analysis Working Group (CAWG) Metabolomics Standards Initiative (MSI). *Metabolomics* **2007**, *3*, 211-221, doi:10.1007/s11306-007-0082-2.
6. Ohno, O.; Chiba, T.; Todoroki, S.; Yoshimura, H.; Maru, N.; Maekawa, K.; Imagawa, H.; Yamada, K.; Wakamiya, A.; Suenaga, K.; et al. Halichonines A, B, and C, novel sesquiterpene alkaloids from the marine sponge *Halichondria okadai* Kadota. *Chem. Commun.* **2011**, *47*, 12453-12455, doi:10.1039/c1cc15557a.
7. Kashman, Y.; Zviely, M. New alkylated scalarins from the sponge *Dysidea herbacea*. *Tetrahedron Lett.* **1979**, *20*, 3879-3882, doi:10.1016/S0040-4039(01)95551-0.
8. Shao, Z.Y.; Zhu, D.Y.; Guo, Y.W. Nanjiols A-C, new steroids from the Chinese soft coral *Nephthea bayeri*. *J. Nat. Prod.* **2002**, *65*, 1675-1677, doi:10.1021/np020087x.
9. Jares-Erijman, E.A.; Bapat, C.P.; Lithgow-Bertelloni, A.; Rinehart, K.L.; Sakai, R. Crucigasterins, new polyunsaturated amino alcohols from the Mediterranean tunicate *Pseudodistoma crucigaster*. *J. Org. Chem.* **1993**, *58*, 5732-5737, doi:10.1021/jo00073a036.
10. Ciavatta, M.L.; Manzo, E.; Nuzzo, G.; Villani, G.; Varcamonti, M.; Gavagnin, M. Crucigasterins A–E, antimicrobial amino alcohols from the Mediterranean colonial ascidian *Pseudodistoma crucigaster*. *Tetrahedron* **2010**, *66*, 7533-7538, doi:10.1016/j.tet.2010.07.056.
11. Sugawara, T.; Zaima, N.; Yamamoto, A.; Sakai, S.; Noguchi, R.; Hirata, T. Isolation of sphingoid bases of sea cucumber cerebrosides and their cytotoxicity against human colon cancer cells. *Biosci. Biotechnol. Biochem.* **2006**, *70*, 2906-2912, doi:10.1271/bbb.60318.
12. Cameron, G.M.; Stapleton, B.L.; Simonsen, S.M.; Brecknell, D.J.; Garson, M.J. New sesquiterpene and brominated metabolites from the tropical marine sponge *Dysidea* sp. *Tetrahedron* **2000**, *56*, 5247-5252, doi:10.1016/S0040-4020(00)00434-8.
13. Berrue, F.; Ibrahim, A.; Boland, P.; Kerr, R.G. Newly isolated marine *Bacillus pumilus* (SP21): A source of novel lipoamides and other antimicrobial agents. *Pure Appl. Chem.* **2009**, *81*, 1027-1031, doi:10.1351/pac-con-08-09-25.
14. Jiang, W.; Zhou, W.; Uchida, H.; Kikumori, M.; Irie, K.; Watanabe, R.; Suzuki, T.; Sakamoto, B.; Kamio, M.; Nagai, H. A new lyngbyatoxin from the Hawaiian cyanobacterium *Moorea producens*. *Mar. Drugs* **2014**, *12*, 2748-2759, doi:10.3390/md12052748.
15. Irie, K.; Funaki, A.; Koshimizu, K.; Hayashi, H.; Arai, M. Structure of blastmycetin E, a new teleocidin-related compound, from *Streptoverticillium blastmyceticum*. *Tetrahedron Lett.* **1989**, *30*, 2113-2116, doi:10.1016/S0040-4039(01)93726-8.
16. Lin, K.; Yang, P.; Yang, H.; Liu, A.H.; Yao, L.G.; Guo, Y.W.; Mao, S.C. Lysophospholipids from the Guangxi sponge *Spirastrella purpurea*. *Lipids* **2015**, *50*, 697-703, doi:10.1007/s11745-015-4028-6.
17. Ikeda, T.; Yasuda-Kamatani, Y.; Minakata, H.; Kenny, P.T.M.; Nomoto, K.; Muneoka, Y. *Mytilus*-inhibitory peptide analogues isolated from the ganglia of a pulmonate mollusc, *Achatina fulica*. *Comp. Biochem. Physiol.* **1992**, *101*, 245-249, doi:10.1016/0742-8413(92)90268-C.
18. Matsuno, T.; Ookubo, M. The first isolation and identification of fritschiellaxanthin from a crab *Sesarma haematocheir* (Akategani in Japanese). *Bull. Jpn. Soc. Sci. Fish.* **1982**, *48*, 653-659, doi:10.2331/suisan.48.653.
19. Wan, F.; Erickson, K.L. Serinol-derived malyngamides from an Australian cyanobacterium. *J. Nat. Prod.* **1999**, *62*, 1698-1699, doi:10.1021/np990291t.
20. Sugawara, T.; Tanaka, A.; Nagai, K.; Suzuki, K.; Okada, G. New members of the trichothecene family. *J. Antibiot.* **1997**, *50*, 778-780, doi:10.7164/antibiotics.50.778.

21. Garrido, L.; Zubia, E.; Ortega, M.J.; Naranjo, S.; Salva, J. Obscuraminols, new unsaturated amino alcohols from the tunicate *Pseudodistoma obscurum*: Structure and absolute configuration. *Tetrahedron* **2001**, *57*, 4579-4588, doi:10.1016/S0040-4020(01)00372-6.
22. Fujiwara, Y.; Maoka, T.; Ookubo, M.; Matsuno, T. Crassostreaxanthins A and B, novel marine carotenoids from the oyster *Crassostrea gigas*. *Tetrahedron Lett.* **1922**, *33*, 4941-4944, doi:10.1016/S0040-4039(00)61240-6.
23. Benveniste, J.; Henson, P.M.; Cochrane, C.G. Leukocyte-dependent histamine release from rabbit platelets. The role of IgE, basophils, and a platelet-activating factor. *J. Exp. Med.* **1972**, *136*, 1356-1377, doi:10.1084/jem.136.6.1356.
24. Nonaka, K.; Tsukiyama, T.; Okamoto, Y.; Sato, K.; Kumasaka, C.; Yamamoto, T.; Maruyma, F.; Yoshikawa, H. New milbemycins from *Streptomyces hygroscopicus* subsp. *aureolacrimosus*: Fermentation, isolation and structure elucidation. *J. Antibiot.* **2000**, *53*, 694-704, doi:10.7164/antibiotics.53.694.
25. Imperatore, C.; Aiello, A.; D'Aniello, F.; Luciano, P.; Vitalone, R.; Meli, R.; Raso, G.M.; Menna, M. New bioactive alkyl sulfates from Mediterranean tunicates. *Molecules* **2012**, *17*, 12642-12650, doi:10.3390/molecules171112642.
26. Carroll, A.R.; Scheuer, P.J. Four beta-alkylpyridines from a sponge. *Tetrahedron* **1990**, *46*, 6637-6644, doi:10.1016/S0040-4020(01)87855-8.
27. Rinehart, K.L.; Kishore, V.; Bible, K.C.; Sakai, R.; Sullins, D.W.; Li, K.M. Didemnins and tunichlorin: Novel natural products from the marine tunicate *Trididemnum solidum*. *J. Nat. Prod.* **1988**, *51*, 1-21, doi:10.1021/np50055a001.
28. Tomiyama, T. The chemical composition of tunny liver oil. *Bull. Chem. Soc. Jpn.* **2014**, *9*, 141-147, doi:10.1080/03758397.1933.10857049.
29. Aguilar-Martinez, M.; Liaaen-Jensen, S. Animal carotenoids. 9. Trikentriorhodin. *Acta Chem. Scand. B* **1974**, *10*, 1247-1248, doi:10.3891/acta.chem.scand.28b-1247.
30. Zbarskiĭ, V.B.; Lazhko, E.I.; Pomanova, N.P.; Fomicheva, E.V.; Saburova, T.P. Rubomycins M and N-new anthracycline antibiotics. *Russ. J. Bioorg. Chem.* **1991**, *17*, 1698-1701.
31. Zhao, Q.; Lee, S.Y.; Hong, J.; Lee, C.O.; Im, K.S.; Sim, C.J.; Lee, D.S.; Jung, J.H. New acetylenic acids from the marine sponge *Stelletta* species. *J. Nat. Prod.* **2003**, *66*, 408-411, doi:10.1021/np020440z.
32. Gutierrez-Cepeda, A.; Fernandez, J.J.; Norte, M.; Montalvao, S.; Tammela, P.; Souto, M.L. Acetate-derived metabolites from the brown alga *Lobophora variegata*. *J. Nat. Prod.* **2015**, *78*, 1716-1722, doi:10.1021/acs.jnatprod.5b00415.
33. Lopez, A.; Gerwick, W.H. Two new icosapentaenoic acids from the temperate red seaweed *Ptilota filicina* J. Agardh. *Lipids* **1987**, *22*, 190-194, doi:10.1007/BF02537301.
34. Watanabe, N.; Yamamoto, K.I.; Ihshikawa, H.; Yagi, A.; Sakata, K.; Brinen, L.S.; Clardy, J. New chlorophyll-a-related compounds isolated as antioxidants from marine bivalves. *J. Nat. Prod.* **1993**, *56*, 305-317, doi:10.1021/np50093a001.
35. Watanabe, K.; Tsuda, Y.; Hamada, M.; Omori, M.; Mori, G.; Iguchi, K.; Naoki, H.; Fujita, T.; Van Soest, R.W. Acetylenic strongylodiols from a *Petrosia* (*Strongylophora*) Okinawan marine sponge. *J. Nat. Prod.* **2005**, *68*, 1001-1005, doi:10.1021/np040233u.
36. Pearce, A.N.; Appleton, D.R.; Babcock, R.C.; Copp, B.R. Distomadines A and B, novel 6-hydroxyquinoline alkaloids from the New Zealand ascidian, *Pseudodistoma aureum*. *Tetrahedron Lett.* **2003**, *44*, 3897-3899, doi:10.1016/s0040-4039(03)00831-1.
37. Osuka, A.; Wada, Y.; Shinoda, S. Covalently linked pyropheophorbide dimers as models of the special pair in the photosynthetic reaction center. *Tetrahedron* **1996**, *52*, 4311-4326, doi:10.1016/0040-4020(96)00131-7.
38. Neunlist, S.; Rohmer, M. A novel hopanoid, 30-(5'-adenosyl)hopane, from the purple non-sulphur bacterium *Rhodopseudomonas acidophila*, with possible DNA interactions. *Biochem. J.* **1985**, *228*, 769-771, doi:10.1042/bj2280769.
